# Breaking the Tumor Chronic Inflammation Balance with a Programmable Release and Multi‐Stimulation Engineering Scaffold for Potent Immunotherapy

**DOI:** 10.1002/advs.202401377

**Published:** 2024-05-17

**Authors:** Xiuqi Liang, Xinchao Li, Rui Wu, Tao He, Furong Liu, Lu Li, Yi Zhang, Songlin Gong, Miaomiao Zhang, Xiaorong Kou, Tao Chen, Yanjie You, Meiling Shen, Qinjie Wu, Changyang Gong

**Affiliations:** ^1^ Department of Biotherapy Cancer Center and State Key Laboratory of Biotherapy West China Hospital Sichuan University Chengdu 610041 China; ^2^ Department of Anesthesiology Shengjing Hospital of China Medical University Shenyang China; ^3^ Department of Gastroenterology People's Hospital of Ningxia Hui Autonomous Region Yinchuan 750002 China

**Keywords:** cancer immunotherapy, engineering scaffold, multi‐stimulation, programmable release, tumor chronic inflammation

## Abstract

Tumor‐associated chronic inflammation severely restricts the efficacy of immunotherapy in cold tumors. Here, a programmable release hydrogel‐based engineering scaffold with multi‐stimulation and reactive oxygen species (ROS)‐response (PHOENIX) is demonstrated to break the chronic inflammatory balance in cold tumors to induce potent immunity. PHOENIX can undergo programmable release of resiquimod and anti‐OX40 under ROS. Resiquimod is first released, leading to antigen‐presenting cell maturation and the transformation of myeloid‐derived suppressor cells and M2 macrophages into an antitumor immune phenotype. Subsequently, anti‐OX40 is transported into the tumor microenvironment, leading to effector T‐cell activation and inhibition of Treg function. PHOENIX consequently breaks the chronic inflammation in the tumor microenvironment and leads to a potent immune response. In mice bearing subcutaneous triple‐negative breast cancer and metastasis models, PHOENIX effectively inhibited 80% and 60% of tumor growth, respectively. Moreover, PHOENIX protected 100% of the mice against TNBC tumor rechallenge by electing a robust long‐term antigen‐specific immune response. An excellent inhibition and prolonged survival in PHOENIX‐treated mice with colorectal cancer and melanoma is also observed. This work presents a potent therapeutic scaffold to improve immunotherapy efficiency, representing a generalizable and facile regimen for cold tumors.

## Graphical abstract

The delicate and ever‐changing nature of tumor‐associated chronic inflammation adversely affects anticancer immunity efficiency. Herein, a programmable release hydrogel‐based engineering scaffold with multi‐stimulation and reactive oxygen species (ROS)‐response (PHOENIX) is designed to break the chronic inflammatory balance in cold tumors, resulting in enhanced immunotherapy efficiency and represent a generalizable and facile regimen for cold tumor treatment.

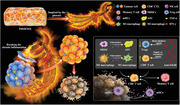

## 1 Introduction

With the advantages of high specificity and low toxicity, cancer immunotherapy has emerged as a novel strategy for malignant tumor therapy.^[^ ^1^ ^]^ However, immunotherapy strategies for cold tumors, such as immune checkpoint blockade (ICB) therapies and cancer vaccines, are often constrained by scarce tumor‐infiltrating lymphocytes (TILs), low tumor mutational burden (TMB), weak antigen‐presenting cell (APC) maturation, and immunosuppressive factors, such as myeloid‐derived suppressor cells (MDSCs) and M2 macrophages in the tumor microenvironment (TME), leading to insufficient immunotherapy efficiency.^[^ ^2^ ^]^ In this regard, combination immunotherapy strategies, such as chemoimmunotherapy, radioimmunotherapy, photoimmunotherapy, and postoperative immunotherapy, have been extensively studied to first trigger tumor‐associated antigens (TAAs) in situ by chemotherapy, radiotherapy, photothermal therapy, or surgery and subsequently restore effectors T cells (Teffs) function through inhibitory ICB antibodies.^[^ ^3^ ^]^ Although achievements have been made, the efficacy of these programs in dealing with tumor‐associated chronic inflammation is still limited.^[^ ^4^ ^]^ Therefore, it is highly desirable to develop a novel therapeutic strategy to overcome chronic inflammation‐induced insufficient immunotherapy in cold tumors.

Tumor‐associated chronic inflammation is closely associated with the occurrence and development of multiple malignancies, as well as with the effectiveness of antitumor immunotherapy.^[^ ^5^ ^]^ Chronic inflammation associated with infections or autoimmune diseases, such as *Helicobacter pylori* hepatitis B (HBV) virus infections, can significantly increase the risk of tumorigenic events.^[^ ^6^ ^]^ In addition, chronic inflammation modulates the adaptive immune responses by producing anti‐inflammatory lipid mediators and cytokines, such as IL‐10, TGF‐β, and the epidermal growth factor (EGF) family,^[^ ^5^, ^7^ ^]^ which further leads to the production of regulatory T (Treg) and B cells, the exhaustion of effector T cells, the inhibition of antitumor immune activation for a long time and the eventual shift of antitumor immunity to protumorigenic inflammation.^[^ ^8^ ^]^ Chronic inflammation associated with local immunosuppression will further limit the effect of antitumor immunotherapy, especially in cold tumors with less immune cell infiltration.^[^ ^9^ ^]^ Thus, breaking the balance of chronic inflammation in cold tumors and inducing inflammation in the immune activation arm might be a promising strategy to drive the inflammatory response against cold tumors.

Here, we demonstrate a programmable release hydrogel‐based engineering scaffold with multi‐stimulation and reactive oxygen species (ROS)‐response (abbreviated as PHOENIX) to break the chronic inflammatory balance in cold tumors. After local administration, PHOENIX can undergo programmable release of resiquimod (R848) and anti‐OX40 (aOX40) in the presence of abundant ROS: R848 is first released, causing a reduction of inhibitory MDSCs and TAMs, favoring breaking the chronic inflammation; aOX40 is subsequently transported into the TME and accelerates T‐cell activation and expansion even in the absence of TAAs, eliciting T cell‐mediated killing and generating neoantigens and leading to the reversal of tumor‐associated chronic inflammation. The neoantigen with R848 excites APCs and further intensifies the infiltration of lymphocytes. Through a series of cascade amplification reactions, PHOENIX breaks the balance of chronic inflammation in cold tumors, thus inducing a potent immune response. We applied PHOENIX to a representative cold triple‐negative breast cancer (TNBC) model and found that PHOENIX eradicated 80% of primary TNBC tumor growth and inhibited 60% of tumor metastasis. In addition, PHOENIX effectively inhibited TNBC tumor rechallenge by electing a robust long‐term antigen‐specific immune response. Furthermore, in other cold tumor models, including melanoma and colorectal cancer models, PHOENIX effectively inhibited tumor growth and prolonged mouse survival. This work demonstrated that breaking the chronic inflammation balance in the TME by an engineering scaffold with ROS‐activated programmed release and multi‐stimulation can be a potent strategy for improving immunotherapy efficiency, representing a generalizable and facile regimen for cold tumor treatment. Our findings also suggested that engineered scaffolds have great potential for application in cancer immunotherapy.

## 2 Results and Discussion

### 2.1 Preparation and Characterization of PHOENIX

**Figure 1:**
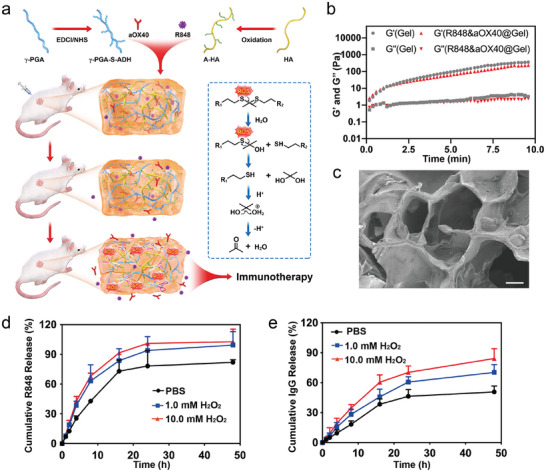
Preparation and characterization of the engineering scaffold. A) The scheme was shown that R848 and aOX40 were programmed released in local tumors with abundant ROS. B) Rheological behaviors of the Gel and R848&aOX40@Gel. C) Representative SEM image of the hydrogel (scale bar, 80 µm). D,E) In vitro cumulative release profiles of R848 D) and IgG (E) from the scaffold in PBS, PBS with 1 mM H_2_O_2_, and PBS with 10 mM H_2_O_2_.

We employed the chemical crosslinking between aldehyde hyaluronic acid (A‐HA) and poly N″‐(3‐((2‐((3‐hydrazineyl‐3‐oxopropyl) thio) propan‐2‐yl) thio) propanoyl)−2‐(methylamino)−5‐oxohexanehydrazide (γ‐PGA‐S‐ADH) to generate a ROS‐responsive scaffold (**Figure** **1**a; Figure S1, Supporting Information). A‐HA was synthesized and characterized by Fourier transform infrared (FTIR) spectroscopy and *^1^H*‐NMR (Figure S2, Supporting Information). The oxidation degree of A‐HA was measured by hydroxylamine hydrochloride, and the theoretical oxidation degree and the actual oxidation degree measurement results are shown in Table S1 (Supporting Information). The average oxidation degree of A‐HA is ≈45.83%. γ‐PGA‐S‐ADH contains a ROS‐sensitive linker, synthesized via a thioketal diol reaction and amidation reaction, and confirmed by *^1^H*‐NMR (Figure S3A–C, Supporting Information). To detect the ROS responsiveness of the scaffold, we exposed the A‐HA/γ‐PGA‐S‐ADH gel to H_2_O_2_ and found that the A‐HA/γ‐PGA‐S‐ADH gel could be oxidized, causing the dissociation of the scaffold (Figure S3D, Supporting Information). Next, the A‐HA/γ‐PGA‐S‐ADH scaffold was analyzed through rheological testing. The storage modulus (G′) rapidly increased and exceeded the loss modulus (G″) after the addition of A‐HA to the γ‐PGA‐S‐ADH solution, which demonstrated that a network between γ‐PGA‐S‐ADH and A‐HA was formed (Figure S3E, Supporting Information). The A‐HA/γ‐PGA‐S‐ADH scaffold was able to undergo a temperature‐dependent phase transition. With increasing temperature, G″ changed faster than G″ and gradually exceeded G″, and gelation occurred at ≈17 °C (Figure S3F, Supporting Information). The morphology of the dried scaffold could be observed by scanning electron microscopy (SEM), which indicated the porous network structure in the engineering scaffold (Figure 1c). The toxicity of the A‐HA/γ‐PGA‐S‐ADH hydrogel in normal cells was further examined. MTT assays showed that the gel extract solution had no noticeable side effects on L929 or NIH 3T3 cells (Figure S4A–F, Supporting Information). Additionally, gelation and degradation behaviors were assessed in healthy BALB/c mice (Figure S4G, Supporting Information). After 21 days of implantation, most gels had degraded, demonstrating the excellent biodegradability of the A‐HA/γ‐PGA‐S‐ADH scaffold.

After evaluating the properties of the A‐HA/γ‐PGA‐S‐ADH scaffold, R848 and aOX40 were encapsulated in the scaffold to form PHOENIX (Figure 1a). We examined the rheological behaviors of R848@Gel, aOX40@Gel, and PHOENIX (R848&aOX40@Gel). We found that the rheological properties of these scaffolds were similar to those of Gel alone (Figure 1b), suggesting that the gelation properties did not noticeably change after drug loading. The release behaviors of R848 and aOX40 were analyzed by high‐performance liquid chromatography (HPLC) and ultraviolet spectrophotometry, respectively. To test ROS sensitivity, PHOENIX was placed in phosphate‐buffered saline (PBS), PBS containing 1.0 mM H_2_O_2_, or PBS containing 10.0 mM H_2_O_2_. Those in the H_2_O_2_ showed a faster rate than release behaviors in PBS, suggesting an H_2_O_2_ concentration‐dependent manner. Besides, the release rates of R848 were greater than those of aOX40 (Figure 1d,e), confirming the programmed release behaviors of the engineering scaffold, which favors the induction of cascade‐amplification immune effects in tumors.

### 2.2 R848@Gel Drives the Balance of Chronic Inflammation to the Immune Activation Arm

**Figure 2:**
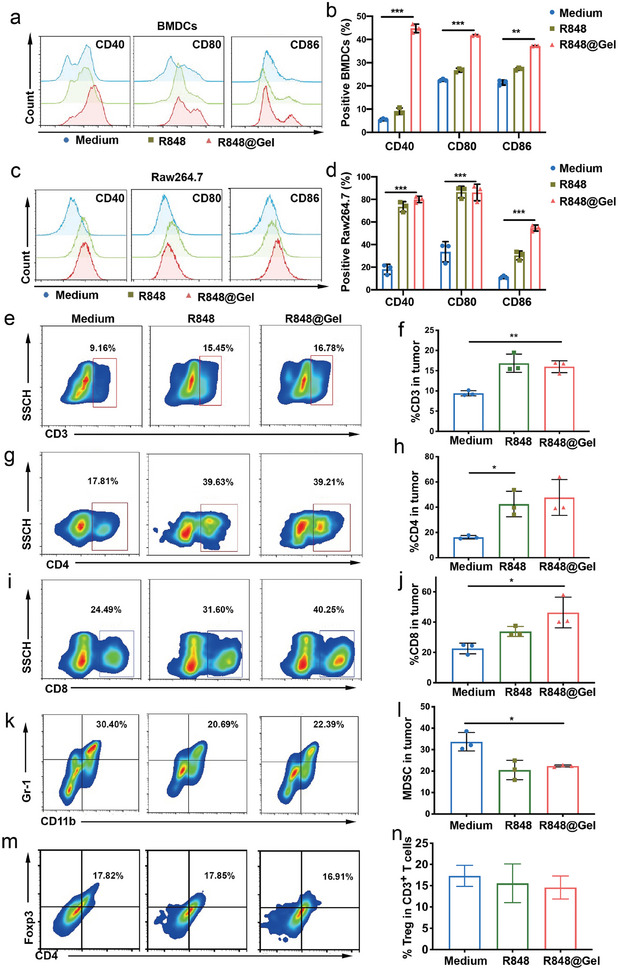
R848@Gel drives the balance of chronic inflammation to the immune activation arm. A,B) BMDCs were cultured and incubated with free R848 and R848@Gel, and then analyzed by flow cytometry (n = 3 per group). C,D) Raw 264.7 cells were cultured and incubated with free R848 and R848@Gel, and then analyzed by flow cytometry (n = 3 per group). E–N) 4T1 tumor‐bearing mice were treated with free R848 or R848@Gel and analyzed by flow cytometry (n = 3, biologically independent samples). Representative flow cytometric plots of T‐cell infiltration in local tumors (E), CD4^+^ T cells (G), CD8^+^ T cells (I), MDSCs (CD11b^+^ Gr‐1^+^) (K), and Tregs (CD3^+^CD4^+^Foxp3^+^). The data are shown as the mean ± SD. Statistical significance was calculated by one‐way analysis of variance (ANOVA) with Tukey's post‐test. ^*^ *p* < 0.05; ^**^ *p* < 0.01; ^***^ *p* < 0.001.

Given that R848 was first released from PHOENIX, the effect of R848@Gel on the balance of chronic inflammation was first explored. We preliminarily explored the impact of engineering scaffolds on APCs. BMDCs were respectively incubated with R848@Gel and free R848, and CD40, CD80, and CD86 expression was analyzed by flow cytometry. As expected, the expression of CD40, CD80, and CD86 on BMDCs was upregulated (**Figure** **2**a,b), suggesting the immunostimulatory effects of R848@Gel. It is well known that TLR7/8 is expressed in DCs, macrophages, and MDSCs. TLR agonists can polarize TAMs into an antitumor phenotype for macrophages by activating the NF‐κB signaling pathway.^[^ ^10^ ^]^ The upregulation of CD40, CD80, and CD86 demonstrates the antitumor immune polarization of macrophages. Hence, the effect of R848@Gel on costimulatory molecules on the surface of macrophages was then explored. CD40, CD80, and CD86 on Raw 264.7 cells were increased, while CD206 expression was reduced, which indicated a positive transition for further inducing the chronic inflammation balance in the immune activation arm (Figure 2c,d; Figure S5A,B, Supporting Information). R848@Gel also improved the percentage of CD69^+^ NK cells, suggesting that R848@Gel activated NK cells and might directly kill tumor cells in a nonspecific way (Figure S5C,D, Supporting Information).

Next, a TNBC tumor model, as one of the cold tumors, was applied to evaluate the immune activation ability of R848@Gel. We established a 4T1‐bearing mouse model and divided the mice into three groups: PBS, R848, and R848@Gel. Then, we prepared the single‐cell suspensions of tumors and analyzed them by flow cytometry. We found that the free R848 and R848@Gel system increased the percentage of TILs in local tumors (Figure 2e,f), as did both the frequency of CD4^+^ T cells (Figure 2g,h) and the frequency of CD8^+^ T cells (Figure 2i,j), suggesting that R848@Gel can enhance lymphocytic infiltration. The effects of R848@Gel on immunosuppressive cells were also explored. The number of MDSCs was significantly decreased, which is consistent with previous research (Figure 2k,l).^[^ ^11^ ^]^ Compared with those in the NS group, R848, and R848@Gel had no significant effect on the content of Treg cells in the tumor microenvironment (Figure 2m,n). This data demonstrated that R848@Gel could enhance lymphocyte infiltration and inhibit immunosuppressive cell function, which drives the inflammatory response against cold tumors. This finding also urged us to develop a collaborative strategy to inhibit Treg function.

### 2.3 PHOENIX Inhibits Cold Tumor Growth in a Triple‐Negative Breast Cancer Model

**Figure 3:**
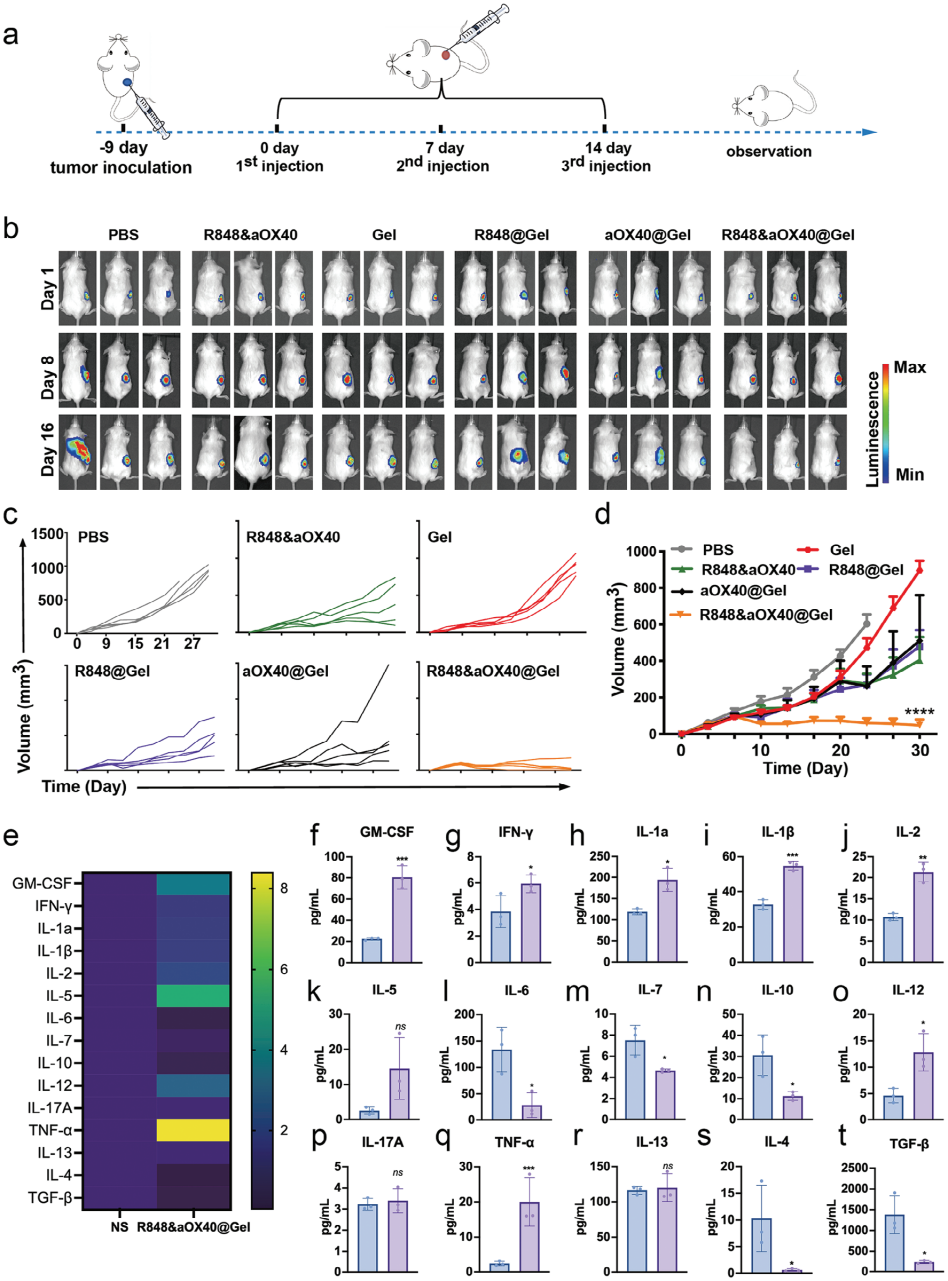
PHOENIX (R848&aOX40@Gel) inhibited TNBC tumor growth in mice. A) Schematic illustration of the TNBC subcutaneous tumor model. B) Bioluminescence imaging of the 4T1 tumors in BALB/c mice. C,D) Individual mouse tumor growth curves (C) and average tumor growth curves (D) in different treatment groups. The average growth curves are shown as mean ± SEM (n = 5, biologically independent samples). Statistical significance was calculated by two‐way ANOVA using the Tukey post‐test. *p*‐value: ^*^ *p* < 0.05; ^**^ *p* < 0.01; ^***^ *p* < 0.001, and ^****^ *p* < 0.0001.). E–T) 4T1 tumor‐bearing mice were administered normal saline or R848&aOX40@Gel three times to analyze cytokine levels in the tumor microenvironment. Heatmap representation of relative cytokine expression in tumors (E) and statistical graphs of cytokine contents were presented (F–T). The data are shown as mean ± SD. Statistical significance was calculated by an unpaired, two‐tailed Student's *t*‐test.

To test whether the engineering scaffold could inhibit cold tumor growth, a TNBC tumor model with a low immunogenic phenotype and abundant chronic inflammation was employed.^[^ ^12^ ^]^ 4T1 tumor‐bearing mice were randomly divided into six groups and treated with different formulations, R848@Gel, R848&aOX40@Gel (PHOENIX), aOX40@Gel, Gel only, free R848+aOX40 and PBS. The R848 dose was 20 µg per mouse, and the aOX40 dose was 8 µg per mouse (**Figure** **3**a). Tumor growth was evaluated by bioluminescence imaging (Figure 3b) and tumor volume curves (Figure 3c,d). As expected, the R848&aOX40@Gel‐treated mice demonstrated adequate inhibition, whereas the gel‐treated mice exhibited no apparent effects. R848@Gel‐treated mice and aOX40@Gel‐treated mice showed a delay in tumor growth. Next, we analyzed tumor curves between the R848&aOX40@Gel group and the R848&aOX40 group and found that the R848&aOX40@Gel group was more remarkable than the free group in inhibiting tumor growth. This observation confirms that the engineering scaffold is beneficial for exerting an antitumor immune response. Notably, 80% of the tumors in the PHOENIX‐treated group were completely regressed over time, suggesting that our strategy could be a powerful means to cure TNBC. The body weight curves showed no detectable changes before and after treatment among all groups (Figure S6, Supporting Information), indicating that the formulations had no visible side effects. To evaluate the effect of PHOENIX on tumor‐associated chronic inflammation, cytokines in the tumor microenvironment of the normal saline (NS)‐treated mice and PHOENIX‐treated mice were detected. Compared with those in the NS group, the contents of anti‐inflammatory cytokines and cancer‐promoting inflammatory cytokines, including IL‐6, IL‐7, IL‐10, IL‐4, and TGF‐β, were significantly reduced after R848&aOX40@Gel treatment. Moreover, the levels of cancer‐inhibiting inflammatory cytokines, such as IFN‐γ, IL‐2, IL‐12, and TNF‐α, were greatly enhanced in the tumor microenvironment (Figure 3e–t).

### 2.4 PHOENIX Breaks the Balance of Chronic Inflammation in Cold Tumors by Evoking a Cascading Immune Response

**Figure 4:**
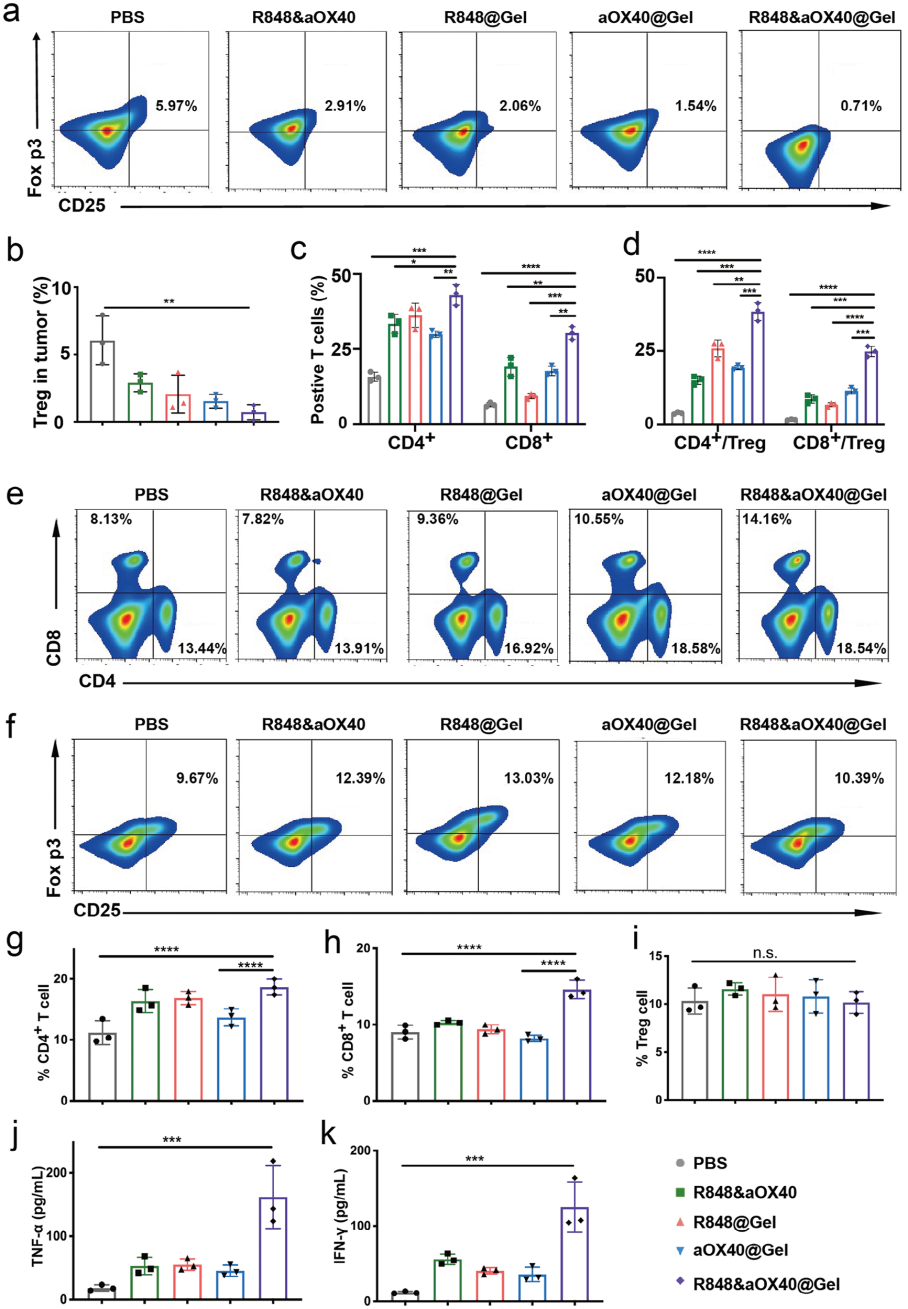
PHOENIX breaks the balance of chronic inflammation in cold tumors by evoking a cascading immune response. A,B) After being administered with R848@Gel, free R848, and aOX40, R848&aOX40@Gel, or aOX40@Gel, the representative flow cytometric plots of Tregs in local tumors were shown. C) The percentages of tumor‐infiltrating CD4^+^ T cells and CD8^+^ CTLs in the mentioned groups were indicated. D) CD4^+^ T cells to Treg ratios and CD8^+^CTLs to Treg ratios were presented (n = 3, biologically independent samples. The data are shown as the mean ± SD. Statistical significance was calculated by one‐way ANOVA using the Tukey post‐test. *p*‐values: ^*^ *p* < 0.05; ^**^ *p* < 0.01; ^***^ *p* < 0.001, and ^****^ *p* < 0.0001.) E–I) After administered with R848@Gel, free R848, and aOX40, R848&aOX40@Gel or aOX40@Gel, the percentages of CD4^+^ T cells and CD8^+^ T cells in the spleen (E), (G), and (H) and the percentage of Tregs (CD4^+^ CD25^+^ and Foxp3^+^) in the spleen (F) and (I) were presented (n = 3 per group). J,K) The secretions of TNF‐α (J) and IFN‐γ (K) in the serum were analyzed by ELISA (n = 3 per group). The data are shown as mean ± SD. Statistical significance was calculated by one‐way ANOVA using the Tukey post‐test. *p*‐value: ^*^ *p* < 0.05; ^**^ *p* < 0.01; ^***^ *p* < 0.001, and ^****^ *p* < 0.0001.

The immunocellular composition of the 4T1 tumors was further analyzed to evaluate whether the chronic inflammation balance was broken down. Tumors were harvested, and single‐cell suspensions were prepared for flow cytometry. The percentages of Tregs (CD3^+^ CD25^+^ and Foxp3^+^) were ≈5.97%, 2.06%, 2.91%, 0.71%, and 1.54% in the PBS, R848@Gel, free, R848&aOX40@Gel and aOX40@Gel groups, respectively, suggesting that the Treg content in R848&aOX40@Gel‐treated mice was lower than that in the other groups (**Figure** **4**a,b). Moreover, R848&aOX40@Gel‐treated mice suggested higher CD8^+^ and CD4^+^T cell infiltration in the tumor than did the other groups (Figure 4c). Notably, aOX40@Gel‐treated mice also had lower Treg levels, confirming that Treg phenotypes were suppressed by activating OX40 pathways. In addition, the ratios of CD8^+^ and CD4^+^ T cells to immunosuppressive Tregs were analyzed. As shown in Figure 4d, the ratios of CD8^+^ and CD4^+^T cells to Tregs in R848&aOX40@Gel‐treated mouse tumors were 15.73‐fold and 10.03‐fold greater than those in the PBS group, respectively. In addition, we measured the percentage of DCs in tumors and found a higher rate of R848&aOX40@Gel treated mice than others (Figure S7, Supporting Information). These results demonstrated that our strategy reshaped the tumor immune microenvironment and elicited robust T‐cell‐mediated cascade amplification, leading to an imbalance in chronic inflammation.

The systematic immune response was explored by analyzing immune cells in the spleen and cytokine secretion by immune‐related cells in the serum. The CD8^+^ T‐cell population and CD4^+^ T‐cell population in the R848&aOX40@Gel‐treated group were higher than others (Figure 4e), which were 1.62‐fold and 1.67‐fold greater than those in the PBS group, respectively (Figure 4g,h). However, Treg levels demonstrated a negligible difference in all groups (Figure 4f,i). Then, TNF‐α and IFN‐γ secretions in the plasma were measured. As shown in Figure 4j,k, the contents of TNF‐α and IFN‐γ in the R848&aOX40@Gel group were dramatically increased, indicating robust systematic antitumor immune responses. The significant impact on primary cold tumors inspired us to apply this engineering scaffold system for distant metastasis and recurrence.

### 2.5 PHOENIX Protects 100% of the Mice Against TNBC Tumor Rechallenge by Potently Inducing a Long‐Term Antigen‐Specific Immune Response

**Figure 5:**
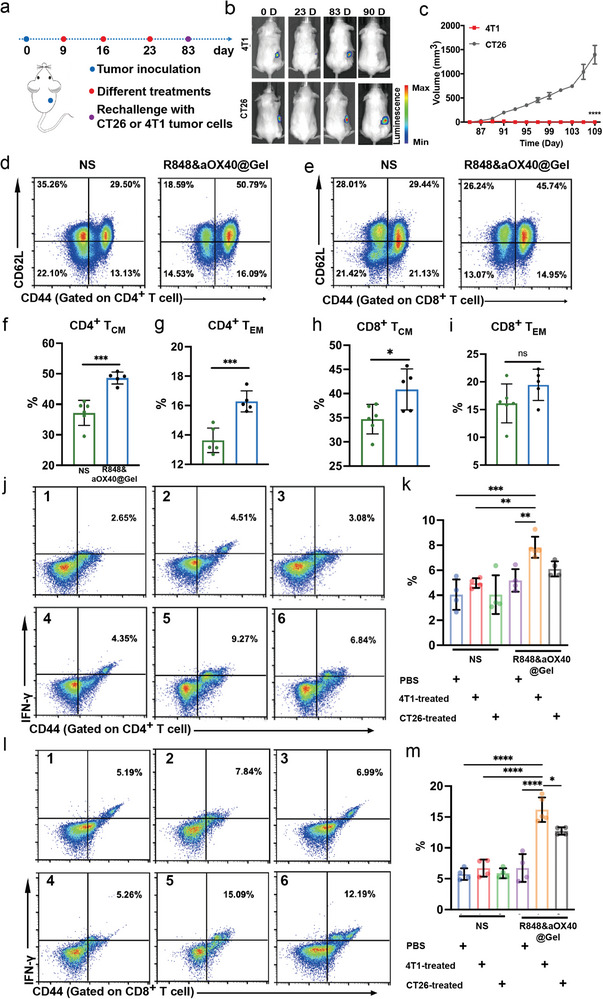
PHOENIX protects 100% of the mice against TNBC tumor rechallenge by potently inducing a long‐term antigen‐specific immune response. A) Schematic illustration of the TNBC tumor recurrence model. B) In vivo bioluminescence images of the rechallenged tumor growth in different groups were shown (n = 3, biologically independent samples). C) Recurrence tumor curves after rechallenge with 4T1 or CT26 cells were shown (n = 3, biologically independent samples). D–I) 4T1 tumor‐bearing mice were treated with NS or R848&aOX40@Gel in the local tumor. After 21 days, the single‐cell suspensions from the spleen were analyzed by flow cytometry. Representative flow cytometric images and percentages of CD4^+^ CD44^+^ CD62L^+^ T cells and CD4^+^ CD44^+^ CD62L^−^ T (D,F–G), and CD8^+^ CD44^+^ CD62L^+^ T cells and CD8^+^ CD44^+^ CD62L^−^ T cells were shown (E,H–I). The data are shown as mean ± SD. Statistical significance was calculated by an unpaired, two‐tailed Student's *t*‐test. (J–M) Single‐cell suspensions from the spleen were cocultured with X‐ray‐treated 4T1 and X‐ray‐treated CT26 tumor cells, respectively. Representative flow cytometric images and percentages of CD44^+^ IFN‐γ^+^ T cells gated on CD 4^+^ T cells J,K) and CD44^+^ IFN‐γ^+^ T cells gated on CD 8^+^ T cells L,M) (group 1, NS‐PBS‐treated; group 2, NS‐4T1‐treated; group 3, NS‐CT26‐treated; group 4, R848&aOX402Gel‐PBS‐treated; group 5, R848&aOX402Gel‐4T1‐treated; group 6, R848&aOX402Gel‐CT26‐treated). The data are shown as mean ± SD. Statistical significance was calculated by one‐way ANOVA using the Tukey post‐test. *p‐*value: ^*^ *p* < 0.05; ^**^ *p* < 0.01; ^***^ *p* < 0.001, and ^****^ *p* < 0.0001.

We further evaluated the effects of PHOENIX on cold tumor recurrence. Notably, the inflammatory effect of reinoculation on tumor formation in the recurrence model differed from that in the orthotopic model. 4T1 cured mice after R848&aOX40@Gel‐treatment were re‐challenged with 4T1 cells or CT26 cells on day 83 (**Figure** **5**a). The growth of distant tumors in the two groups (4T1‐treated group and CT26‐treated group) was then observed by bioluminescence imaging and tumor volume curves. On the 90^th^ day, mice treated with 4T1 had no fluorescence signals at the injection site, while mice treated with CT26 showed clear fluorescence signals (Figure 5b), which indicated that an antigen‐specific memory response had formed. We continued to measure the tumor growth volume and confirmed that the 4T1‐treated group had complete inhibition (Figure 5c).

To further evaluate the mechanism of antitumor immune memory responses generated by PHOENIX, we established a 4T1 tumor‐bearing mouse model, obtained spleen lymphocytes 3 weeks after normal saline and R848&aOX40@Gel treatments, and analyzed the memory T‐cell subtypes and activation of memory T cells after restimulation with different tumor antigens. As shown in Figure 5d–m, compared with the NS group, the contents of effector memory T cells (T_EM_) and central memory T cells (T_CM_) in the spleens of mice treated with R848&aOX40@Gel were significantly enhanced. Specifically, the percentages of CD4^+^ central memory T cells, CD4^+^ effector memory T cells, CD8^+^ central memory T cells, and CD8^+^ effector memory T cells in the R848&aOX40@Gel‐treated group were increased by 11.64%, 2.66%, 6.14%, and 3.33%, respectively (Figure 5d–i). The obtained splenic T lymphocytes were also coincubated with different tumor‐associated antigens, including X‐ray‐treated 4T1 cells and X‐ray‐treated CT26 cells. As shown in Figure 5j–m, spleen lymphocytes obtained from NS‐treated mice exhibited weak memory T‐cell‐mediated immune killing, regardless of which tumor‐associated antigens they faced again. Still, the R848&aOX40@Gel group showed a positive result. The percentages of CD44^+^ IFN‐γ^+^ T cells among CD4^+^ T cells and CD8^+^ T cells in the R848&aOX40@Gel‐treated groups were dramatically increased after stimulation with X‐ray‐treated 4T1 cells. In contrast, the contents of CD44^+^ IFN‐γ^+^ T cells in the X‐ray‐treated CT26 cell groups were lower than those in the groups treated with the same antigens. T‐cell reactivation seven days after three treatments was also evaluated, and the results indicated that R848&aOX40@Gel elicited an antigen‐specific immune response. As shown in Figure S8 (Supporting Information), both IFN‐γ and TNF‐α secretion in the 4T1‐inhibited group increased. As expected, levels of CD4^+^ CD44^+^ T cells, CD8^+^ CD44^+^ T cells, CD44^+^ IFN‐γ^+^ T cells in CD4^+^ T cells, and CD44^+^ IFN‐γ^+^ T cells in CD8^+^ T cells in 4T1‐treated groups were also increased (Figure S9, Supporting Information). These data evidenced that the cold tumor recurrence was entirely inhibited by forming powerful long‐term immunological memory and antigen‐specific immune responses after treatment with the therapeutic scaffold.

### 2.6 PHOENIX Effectively Inhibits Cold Tumor Metastasis

**Figure 6:**
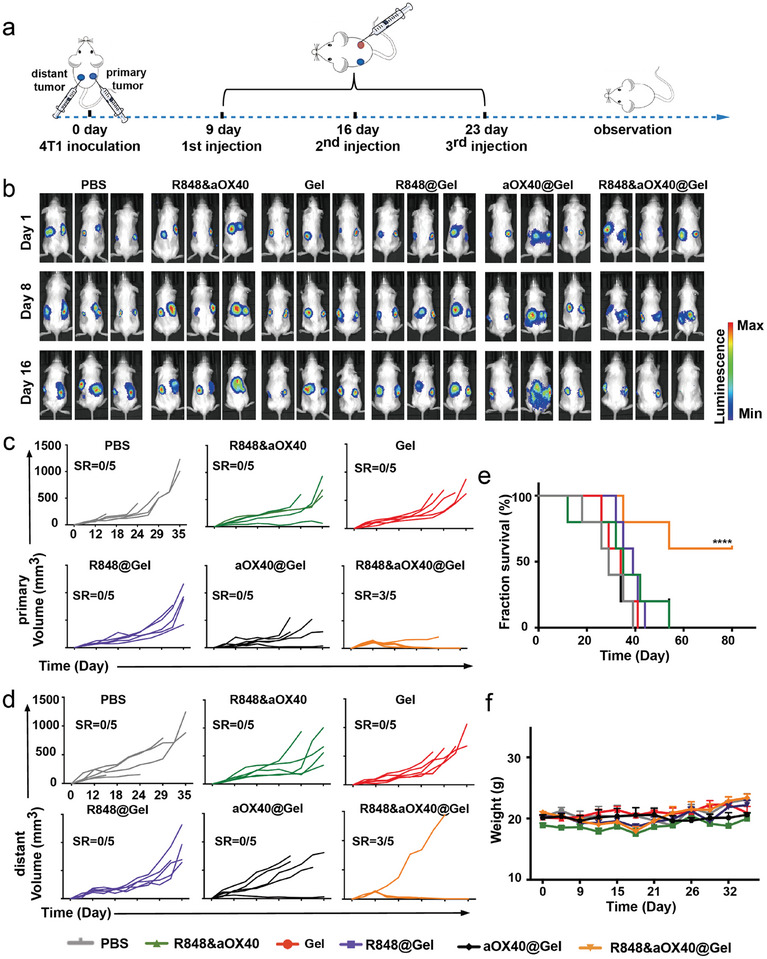
PHOENIX (R848&aOX40@Gel) inhibited 4T1 distant metastasis tumors through a long‐term systemic anticancer immune response. BALB/c mice were inoculated with 4T1 cells in the right and left flanks and then treated with various formulations. A) Schematic illustration of the TNBC tumor metastasis model. B) Bioluminescence imaging of the 4T1 tumors in vivo. C,D) Individual mouse tumor growth curves of the primary tumor (C) and distant tumor (D) (n = 5, biologically independent samples. Statistical significance was calculated by two‐way ANOVA using the Tukey post‐test. *p*‐value: ^*^ *p* < 0.05; ^**^ *p* < 0.01; ^***^ *p* < 0.001, and ^****^ *p* < 0.0001). E) Survival rates of the various treatment groups were presented (n = 5, biologically independent samples). F) Body weight curves of 4T1 tumor‐bearing mice before and after treatment. The data are shown as mean ± SEM. Statistical differences in survival were calculated with the log‐rank test. *p‐*value: ^*^ *p* < 0.05; ^**^ *p* < 0.01; ^***^ *p* < 0.001, and ^****^ *p* < 0.0001.

High metastasis rates are one of the major causes of increased mortality. Hence, we explored whether PHOENIX could inhibit distant metastasis in the cold tumor. 4T1 cells were subcutaneously inoculated into both flanks of mice. After the primary tumor volume reached 100 mm^3^, the therapeutic scaffold was locally injected into the tumor (**Figure** **6**a). Primary and distant tumor growths were evaluated by bioluminescence imaging (Figure 6b) and tumor volume curves (Figure 6C,D). Notably, after treatment with PHOENIX (R848&aOX40@Gel), 60% of distal tumors eventually disappeared within 35 days, which confirmed that a systemic immune response had formed. Treatment with R848@Gel or aOX40@Gel delayed tumor growth, mainly because (1) the primary immune response was insufficient and consequently restricted the systemic immune response and (2) many immunosuppressive cells in metastatic tumors inhibited the systemic immune response. Survival curves of the various treatments were observed. As shown in Figure 6E, 60% of the mice in the R848&aOX40@Gel‐treated group were tumor‐free and survived for >80 days, while the other groups mostly died within 60 days. The body weight curves of the mice were also obtained and showed no noticeable differences before or after receiving the therapeutic scaffold (Figure 6f). These data confirmed that the systemic antitumor immune effect induced by the engineering scaffold was sufficient to reshape the distant tumor microenvironment and inhibit distant metastasis in cold tumors.

### 2.7 PHOENIX has Promising Applications for Other Cold Tumor Types

**Figure 7:**
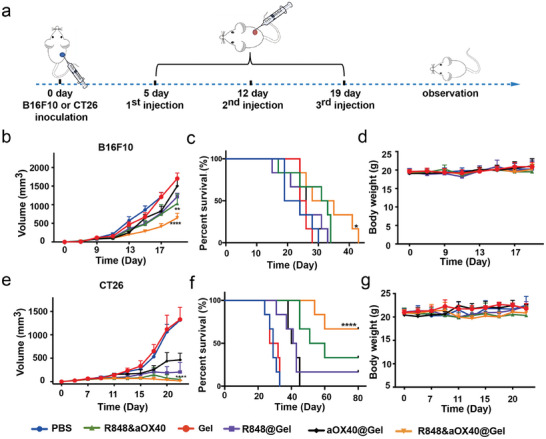
The therapeutic scaffold system is promising for the immunotherapy of other cold tumors. A) Schematic illustration of the B16F10 and CT26 mouse tumor models. B,C) Average tumor growth curves of B16F10 tumor models (B), and survival rates of B16F10 tumor models (C). D) Mouse weight curves of B16F10 before and after treatment (n = 6, biologically independent samples). E,F) Average tumor growth curves of CT26 tumor models (E), and survival rates of CT26 tumor models (F). G) Mouse weight curves of B16F10 before and after treatment (n = 6, biologically independent samples). The data are shown as mean ± SEM. Statistical significance was calculated by two‐way ANOVA using the Tukey post‐test. *p‐*value: ^*^ *p* < 0.05; ^**^ *p* < 0.01; ^***^ *p* < 0.001, and ^****^ *p* < 0.0001. Statistical differences in survival were calculated with the log‐rank test.

The inflammatory response associated with different tumor models varies, and therefore, the efficacy of the same immunotherapy regimen will vary. However, promisingly, OX40 is expressed on the surface of TILs in various tumors, and the receptors of R848 are expressed in both mice and humans.^[^ ^13^ ^]^ These findings support the potential of PHOENIX for the treatment of different cold tumors. To confirm this hypothesis, CT26 murine colon cancer cells, and B16F10 murine melanoma cancer cells were inoculated into BABL/c and C57BL/6 mice, respectively, which are all cold tumors with few TILs and lots of immunosuppressive cells. After the tumor volume reached 100 mm^3^, the mice bearing CT26 tumors or B16F10 tumors were randomly grouped and treated with R848@Gel, R848&aOX40@Gel, aOX40@Gel, Gel only, free R848 and aOX40 (free group), or PBS (**Figure** **7**a). The R848 dose was 20 µg per mouse, the aOX40 amount was 8 µg per mouse, and all gels were 100 µL with 25 mg mL^−1^. Surprisingly, the CT26 tumor model and B16F10 tumor model mice treated with R848&aOX40@Gel exhibited excellent inhibition (Figure 7B,E). Survival rate curves of the B16F10 tumor model mice showed prolonged survival in the R848&aOX40@Gel‐treated group, while the other groups mostly died within 30 days (Figure 7c). A total of 66.67% of the mice in the CT26 tumor model group were tumor‐free and survived for >80 days, while mice treated with R848@Gel or free drugs had 16.67% and 33.33% survival rates, respectively (Figure 7f). Furthermore, the body weights of CT26 tumor‐bearing mice and B16F10 tumor‐bearing mice did not noticeably change after various treatments, demonstrating that the therapeutic scaffold had no obvious toxic effects (Figure 7d,g). These results indicated that PHOENIX has enormous potential for the treatment of multiple cold tumors.

## 3 Conclusion

Here, an engineering scaffold capable of ROS response was applied to encapsulate R848 and aOX40 to form a programmable release hydrogel‐based engineering scaffold with multi‐stimulation and reactive oxygen species (ROS) response (PHOENIX). PHOENIX can react with ROS in the tumor microenvironment and favors breaking down the tumor‐associated chronic inflammation balance. Additionally, R848 and aOX40 were sequentially released from the scaffold. R848 can transform MDSCs and inflammatory M2 macrophages into immune activation subsets, alleviating chronic inflammation. Subsequently, aOX40 could directly activate and expand Teff. By restoring T‐cell function, neoantigens were produced to further excite APCs with R848, enhance the infiltration of lymphocytes, and ultimately cause cascade amplification in cold tumors. The multi‐role of PHOENIX shows excellent performance in the treatment of primary TNBCs and elicits systemic antitumor immune responses that eradicate recurrence and metastasis in mice. Moreover, PHOENIX inhibited the growth of various cold tumors, including B16F10 melanoma and CT26 murine colorectal cancer models. In comparison with conventional chemoimmunotherapy, radioimmunotherapy, or photoimmunotherapy for cold tumors, PHOENIX utilizes “pure” immunotherapy to overcome chronic inflammation in cold tumors, which can avoid the side effects of combination therapies and simplify therapeutic regimens. In PHOENIX, Teff functions can be activated by multiple pathways, including the presentation mechanism of classical MHC molecules and costimulatory factors, and thus can be amplified even in tumors with insufficient tumor mutation antigen burden. Moreover, PHOENIX emphasizes in situ vaccination, which does not prescribe the tumor antigen information, prolongs the retention time of immunomodulators, and protects them from rapid degradation.

Although PHOENIX has been demonstrated to be a superior candidate for enhancing cold tumor therapeutic efficacy, some limitations in this work need further exploration in the future. Here, we demonstrated that breaking the balance of chronic inflammation by shifting the immune cell activation favors a potent antitumor immune response. However, we only applied R848 and aOX40 to implement this strategy. Many immunomodulators, such as indoleamine 2,3‐dioxygenase (IDO) inhibitors, STAT3 inhibitors, TGF‐β inhibitors, CD137 agonists, and CD27 agonists, also have the potential to reshape the tumor immune microenvironment.^[^ ^14^ ^]^ It remains unclear whether these immunomodulators play roles similar to those of R848 and aOX40, and further exploration is needed in the future to expand the strategic application of this work. Moreover, further work is needed to optimize the large‐scale production of the engineering scaffold system for a wider range of cold tumor immunotherapy.

## 4 Experimental Section

### Materials

Methyl 3‐mercaptopropionate and γ‐poly (L‐glutamic acid) macromolecules were purchased from Macklin (Shanghai, China). Sodium hyaluronate was obtained from Bloomage Biotechnology Co. Ltd. (Shandong, China). Acetone, hydrazide hydrate, concentrated sulfuric acid solution, anhydrous Na_2_SO_4_, H_2_O_2_ solution, *N*‐ (3‐dimethtlaminopropyl)‐*N*'‐ethylcarbodilmide hydrochil oride (EDCI) and *N*‐hydroxysuccinimide (NHS) were obtained from Sigma‐Aldrich Co., Ltd. (USA). D‐Luciferin potassium and resiquimod (R848) were purchased from Dalian Meilun Biotechnology Co., Ltd. (Dalian, China). Anti‐mouse OX40 (CD134, aOX40) was obtained from BioXcell (USA). All flow cytometry antibodies were purchased from Biolegend and eBioscience (USA) and are listed in Table S2 (Supporting Information).

### Cell Lines and Animals

The mouse melanoma cell line B16F10, mouse murine colorectal cancer cell line CT26, mouse triple‐negative breast cancer cell line 4T1, mouse fibroblast line L929, and macrophage line Raw 264.7 were obtained from the American Type Culture Collection. NIH 3T3 mouse embryonic fibroblast was obtained from the National Institutes of Health. Luciferase‐expressing mouse breast cancer cell 4T1‐luciferase cells were obtained from iCell Bioscience, Inc. (China). B16F10, CT26, 4T1, and L929 cells were cultured in PRMI 1640 medium supplemented with 10% fetal bovine serum (FBS) and 1% penicillin/streptomycin at 37 °C under 5% CO_2_. NIH 3T3, Raw 264.7, and 4T1‐luciferase cells were maintained in a DMEM medium containing 10% fetal bovine serum (FBS) and 1% penicillin/streptomycin at 37 °C under 5% CO_2_. Female C57BL/6 and BALB/c mice (6 to 8 weeks, 17 to 20 g) were purchased from Dashuo Biotechnology Co. Ltd (Chengdu, China). All animal experiments were performed according to the experimental guidelines of the Animal Experimental Ethics Committee of Sichuan University (2021584A), Chengdu, China.

### Synthesis of γ‐PGA‐S‐ADH and A‐HA

To synthesize dimethyl 3,3′‐(propane‐2,2‐diylbis(sulfanediyl)) dipropionate (ADH), methyl 3‐mercaptopropionate was dissolved in acetone at a concentration of 0.5 mol  L^−1^, and then anhydrous Na_2_SO_4_ and slightly concentrated H_2_SO_4_ were added. The reaction mixture was stirred at 70 °C for 10 h. Next, the Na_2_SO_4_ was removed by filtration. The remaining solution was washed with ethyl acetate, NaHCO_3_ solution, and saturated NaCl solution and concentrated by a rotary evaporator. Finally, the obtained solid was purified by silica gel chromatography and eluted with 92.3% petroleum:7.7% ethyl acetate. The compound was characterized by *^1^H*‐NMR. To synthesize 3,3′‐(propane‐2,2‐diylbis(sulfanediyl)) di (propanehydrazide) (S‐ADH), ADH and N_2_H_4_^.^H_2_O were mixed at a molar ratio of 1:6 in methanol and stirred at room temperature overnight. Then, the product was evaporated, filtered, washed with ice methanol, and dried by a vacuum dryer. To synthesize γ‐PGA‐S‐ADH, γ‐PGA (100 mg, 0.684 mmol) was dissolved in MES buffer. EDCI (157.4 mg, 0.821 mmol) and NHS (94.49 mg, 0.821 mmol) were added for catalysis for 2 h, and then S‐ADH (123.4 mg, 0.684 mmol) was mixed and reacted at room temperature for 48 h. The product was subjected to exhaustive dialysis (MWCO 3500) for dialysis, and the external distilled water phase was changed three times a day for 3 days. Finally, the desired solid was obtained in a vacuum dryer and characterized by *^1^H*‐NMR.

The synthesis and characterization methods for A‐HA were followed by previously reported.^[^ ^15^ ^]^ Briefly, HA (500 mg, 1.26 mmol) was dissolved in deionized water. Sodium periodate (161.69 mg, 0.756 mmol) was added, and the mixture was allowed to react at room temperature for 6 h. Next, ethylene glycol was added to terminate the reaction. The product was purified by exhaustive dialysis (MWCO 3500) for dialysis for 3 days and obtained under vacuum drying. The A‐HA was characterized by FTIR spectroscopy and *^1^H*‐NMR. The oxidation degree of A‐HA was measured by the addition of hydroxylamine hydrochloride.

### Preparation and Characterization of the Engineering Scaffold System

To form the γ‐PGA/ A‐HA scaffold, γ‐PGA‐S‐ADH, and A‐HA were dissolved in PBS buffer at a 25 mg mL^−1^ concentration, respectively. The same volumes of γ‐PGA‐S‐ADH and A‐HA solutions were mixed to form a hydrogel after being fully dissolved. To prepare R848‐ and aOX40‐loaded scaffolds, R848 and aOX40 were fully dispersed in the two matrixes and incorporated at 37 °C. A rheometer was used to test the rheological behavior of the scaffold. To observe the surface morphology, the scaffold was dried and broken in liquid nitrogen and then coated with a thin layer of gold, followed by a scanning electron microscope (SEM) (JSM‐5900LV, JEOL, Japan). To detect the ROS responsiveness of the scaffold, γ‐PGA‐S‐ADH was dissolved in D_2_O and D_2_O containing 10 mM H_2_O_2_ and then incubated at 37 °C for 24 h. Finally, we used the *^1^H*‐NMR spectrum for verification.

### Biodegradation and Cytotoxicity Evaluation of the Scaffold

MTT assay was used for the evaluation of the cytotoxicity of the scaffold.^[^ ^16^ ^]^ Briefly, L929 and NIH 3T3 cells were seeded into 96 well plates at 4 × 10^3^ and 2.5× 10^3^ per well and then incubated with various concentrations of γ‐PGA‐S‐ADH, A‐HA, and the solution after being immersed in PRMI 1640 or DMEM. The cells were incubated for 24 h, then MTT solutions (5 mg mL^−1^, 20 µL per well) were mixed and incubated for another 4 h. Finally, the culture medium was removed and replaced with 150 µL of DMSO. The MTT assay was performed at 560 nm under a microplate reader. To evaluate the degradation behavior of the engineering scaffold in vivo, 200 uL of the scaffold was injected subcutaneously into the right back of mice. Then, the degradation behaviors were observed at particular time points (0, 7, 14, and 21 days).

### Evaluation of the Effect of R848@Gel on APCs

Raw 264.7 cells were cultured and seeded in 24‐well plates at 5×10^4^ per well and then treated with different formulations, R848, R848@Gel, or PBS for 24 h. The obtained cells were washed with PBS twice and stained with PE anti‐mouse CD80 Antibody, APC anti‐mouse CD86 Antibody, or PE anti‐mouse CD40 Antibody (Biolegend, USA) for 30 min on ice. Finally, the cells in PBS were analyzed by flow cytometry (ACEA NovoCyte). For BMDC detection, bone‐marrow‐derived dendritic cells were acquired from the bone marrow of 8‐week‐old C57BL/6 mice and cultured according to a previously established method.^[^ ^17^ ^]^ We incubated BMDCs with various formulations, R848, R848@Gel, and PBS, for 24 h and detected BMDC maturation by flow cytometry. To analyze the effects of R848@Gel on macrophage polarization, Raw 264.7 cells were seeded in 24‐well plates at 5×10^4^ per well, and then, IL‐4 (20 ng mL^−1^) was added to induce M2 macrophages. After 24 h of incubation, R848, R848@Gel, and PBS were added and cultured for another 24 h. The obtained cells were stained with PE anti‐mouse F4/80 Antibody and APC anti‐mouse CD206 Antibody (Biolegend, USA) for 30 min on ice. Finally, the cells in PBS were analyzed by flow cytometry (ACEA NovoCyte). To evaluate the effects of R848@Gel on NK cells, we isolated the spleens of 8‐week‐old C57BL/6 mice, prepared them as single‐cell suspensions, and then cocultured them with different preparations (R848, R848@Gel, and PBS). After 24 h of inhibition, the cells were washed with PBS twice and stained with PE anti‐mouse CD69, PE‐Cy7 anti‐mouse CD3, and PE‐Cy5 anti‐mouse NK1.1 (Biolegend, USA) for 30 min on ice. Finally, the cells in PBS were analyzed by flow cytometry (ACEA NovoCyte).

### In Vivo Evaluation of the Effects of R848@Gel on Infiltrating Immune Cells

4T1 tumor cells were injected subcutaneously into the right‐back of mice at 1 × 10^6^ per mouse. When the tumor volume reached 200 mm^3^, the mice were divided into three groups and peritumorally implanted with the following formulations: PBS, free R848 (20 µg), or R848@Gel (20 µg in 100 uL of scaffold). Forty‐eight hours after treatment, the tumors from the different groups were harvested and prepared as single‐cell suspensions by 2 h incubation in RPMI 1640 medium containing collagenase I and DNase. The following reagents were used to label the suspended single cells: 1) PE‐Cy7 anti‐mouse CD3 Antibody, FITC anti‐mouse CD4 Antibody, and APC anti‐mouse CD8 Antibody; 2) FITC anti‐mouse CD11b Antibody and APC anti‐mouse Gr‐1 Antibody; 3) PE/Cyanine7 anti‐mouse CD3 Antibody, FITC anti‐mouse CD4 Antibody, and PE anti‐mouse Foxp3 Antibody. After 30 min, the cells were washed with PBS twice and analyzed by flow cytometry (ACEA NovoCyte). Notably, after treatment with only the eBioscience Mouse Regulatory T‐Cell Staining Kit (USA), Tregs were stained with a PE anti‐mouse Foxp3 Antibody.

### Antitumor Evaluation In Vivo

Luciferase‐tagged 4T1 cells, CT26 tumor cells, or B16F10 tumor cells were transplanted into the right back of the mice (1 × 10^6^ per mouse). When the tumor volume reached 100 mm^3^, the mice were randomly divided into six groups and treated with different formulations, including R848@Gel (20 µg in 100 µL scaffold), R848&aOX40@Gel (20 µg R848 and 8 µg aOX40 in 100 µL scaffold), aOX40@Gel (8 µg aOX40 in 100 µL scaffold), Gel only (100 µL scaffold), free R848 plus aOX40 (20 µg R848 and 8 µg aOX40 in PBS) and PBS. The tumor size was observed every other day during treatment and calculated by the formula width^2^ × length × 0.5. For 4T1‐luciferase tumor‐bearing mice, the tumor burden was observed by bioluminescence imaging in vivo.

### Cytokine Assay of the Tumor Microenvironment

4T1 tumor‐bearing mice were administered with normal saline or R848&aOX40@Gel three times. After 2 days, the tumors were harvested and triturated for cytokine detection. Normal saline‐treated mice were used as negative controls. Tumor cytokines were determined by Mouse High‐sensitivity bead‐based multiplex assays (MHSTCMAG‐70KPMX, Millipore).^[^ ^18^ ^]^

### Evaluation of the TME and Systemic Antitumor Immune Response

Tumors and spleens were collected from the 4T1‐luciferase tumor model. The flow cytometry was used to analyze various immune cells in tumors and spleens and utilized ELISA to test TNF‐α and INF‐γ secretion.^[^ ^19^ ^]^ The following groups were used to label suspended single cells from tumors: 1) PE/Cyanine7 anti‐mouse CD3 Antibody, FITC anti‐mouse CD4 Antibody, and APC anti‐mouse CD8 Antibody; 2) PE/Cyanine7 anti‐mouse CD3 Antibody, FITC anti‐mouse CD4 Antibody, APC anti‐mouse CD25 Antibody, and PE anti‐mouse Foxp3 Antibody; 3) PE anti‐mouse CD11b Antibody and FITC anti‐mouse CD11c Antibody. The following groups were used to label suspended single cells from the spleen: 1) PE/Cyanine7 anti‐mouse CD3 Antibody, FITC anti‐mouse CD4 Antibody, and APC anti‐mouse CD8 Antibody; 2) PE/Cyanine7 anti‐mouse CD3 Antibody, FITC anti‐mouse CD4 Antibody, APC anti‐mouse CD25 Antibody, and PE anti‐mouse Foxp3 Antibody. ELISA kits were purchased from Biolegend (USA). The representative scatter plots and gating information are shown in Figures S10 and S11 (Supporting Information).

### Tumor Recurrence Model

Luciferase‐tagged 4T1 cells were transplanted into the right back of the mice. On the 9^th^ day, the mice were injected with R848&aOX40@Gel via a dosing regimen, as described above. On the 83^rd^ day, mice with complete tumor regression were divided into two groups: one was rechallenged with luciferase‐tagged 4T1 cells, and the other was rechallenged by luciferase‐tagged CT26 cells. The rechallenged tumor growth was observed every other day during treatment and calculated using the formula width^2^ × length × 0.5. To be more intuitive, the tumor burden was observed by bioluminescence imaging in vivo.

### Evaluation of Immune Memory In Vivo

Spleens were collected from the 4T1‐luciferase tumor recurrence model and prepared into single‐cell suspensions. Next, the suspended single cells were labeled with the following reagents: 1) PE anti‐mouse CD4 Antibody, FTIC anti‐mouse CD44 Antibody, and APC anti‐mouse CD62L Antibody; 2) PE anti‐mouse CD8 Antibody, FTIC anti‐mouse CD44 Antibody, and APC anti‐mouse CD62L Antibody. After 30 min, the cells were washed with PBS twice and analyzed by flow cytometry (ACEA NovoCyte). The representative scatter plots and gating information are shown in Figure S12 (Supporting Information). At the same time, the obtained lymphocytes were seeded in 24‐well plates at 5 × 10^6^ per well, and then X‐ray‐treated 4T1‐luciferase cells or X‐ray‐treated CT26‐luciferase cells were added. After 24 h of incubation, lymphocytes, and culture media were divided for flow cytometry and ELISA detection. Lymphocytes were stained as follows: 1) FITC anti‐mouse CD8a Antibody, APC anti‐mouse CD44 Antibody, PE anti‐mouse IFN‐γ Antibody; 2) FITC anti‐mouse CD4 Antibody, APC anti‐mouse CD44 Antibody, and PE anti‐mouse IFN‐γ Antibody. TNF‐α and INF‐γ ELISA kits were purchased from Biolegend (USA). The representative scatter plots and gating information are shown in Figure S13 (Supporting Information).

### Tumor Metastasis Model

Luciferase‐tagged 4T1 cells were transplanted into both backs of the mice. On the 9^th^ day, the mice were injected with the following formulations, as described above: R848@Gel, R848&aOX40@Gel, aOX40@Gel, Gel only, free R848 plus aOX40, or PBS. The treated tumors (as primary tumors) and untreated tumors (as metastatic tumors) were observed by bioluminescence imaging and calculated using the formula width^2^ × length × 0.5. The survival rates of all the groups were also recorded.

### In Vivo Bioluminescence Imaging of the Mice

Before bioluminescence imaging, the mice were injected with d‐luciferin (Dalian Meilun, China) intraperitoneally. D‐luciferin needs to be dissolved in Dulbeccos PBS (15 mg mL^−1^).^[^ ^20^ ^]^ The injection dose depended on the weight of the mouse (15 µg g^−1^). After 10 min, biological fluorescence images of the mice were collected using an IVIS Spectrum Imaging System (PerkinElmer Ltd., USA), and the exposure time of the imaging acquisition was 2 min. Image analysis was performed using Living Image software (PerkinElmer Ltd., USA).

### Statistical Analysis

All the results were analyzed by GraphPad Prism software (9.0) and were presented as the mean ± SD or mean ± SEM. Unpaired Student's two‐tailed t‐test was performed to determine differences between the two groups. One‐way analysis of variance (ANOVA) analysis and Tukey's post‐test were performed to determine differences between more than two comparisons. Differences in survival benefits were determined using the log‐rank test. Statistical significance is represented by ^*^ *p* < 0.05, ^**^ *p* < 0.01, ^***^ *p* < 0.001, and ^****^ *p* < 0.0001, respectively.

## Conflict of Interest

The authors declare no conflicts of interest.

## Supporting information

Supporting Information

## Data Availability

The data that support the findings of this study are available from the corresponding author upon reasonable request.
