# Supplementary material for: Breaking the Tumor Chronic Inflammation Balance with a Programmable Release and Multi‐Stimulation Engineering Scaffold for Potent Immunotherapy
Source: Adv Sci (Weinh). 2024 May 17;11(28):2401377. doi: 10.1002/advs.202401377 (PMC11267263; doi:10.1002/advs.202401377)
Supplement: Supplementary file 1 — Supporting Information [file ADVS-11-2401377-s001.pdf]

ADVANCED SCIENCE
Open Access

Supporting Information

for *Adv. Sci.*, DOI 10.1002/advs.202401377

Breaking the Tumor Chronic Inflammation Balance with a Programmable Release and Multi-Stimulation Engineering Scaffold for Potent Immunotherapy

*Xiuqi Liang, Xinchao Li, Rui Wu, Tao He, Furong Liu, Lu Li, Yi Zhang, Songlin Gong, Miaomiao Zhang, Xiaorong Kou, Tao Chen, Yanjie You, Meiling Shen, Qinjie Wu and Changyang Gong**

Supporting Information

Breaking the Tumor Chronic Inflammation Balance with a Programmable Release and Multi-stimulation Engineering Scaffold for Potent Immunotherapy

*Xiuqi Liang[1], Xinchao Li[1], Rui Wu[1], Tao He[1], Furong Liu[1], Lu Li[1], Yi Zhang[2], Songlin Gong[1], Miaomiao Zhang[1], Xiaorong Kou[1], Tao Chen[1], Yanjie You[3], Meiling Shen[1], Qinjie Wu[1], Changyang Gong[1]**

[1] Department of Biotherapy, Cancer Center and State Key Laboratory of Biotherapy, West China Hospital, Sichuan University, Chengdu, 610041, China.

[2] Department of Anesthesiology, Shengjing Hospital of China Medical University, Shenyang, China.

[3] Department of Gastroenterology, People's Hospital of Ningxia Hui Autonomous Region, Yinchuan 750002, China.

*To whom correspondence should be addressed (C Gong). E-mail: chygong14@163.com or gongchangyang@scu.edu.cn.

Table S1. Oxidation degree of A-HA.

Table S2. List of antibodies used in flow cytometric analysis.

Fig. S1. Schematic of the engineering scaffold synthesis.

Fig. S2. FTIR spectra and ^{1}H-NMR spectra of HA and A-HA.

Fig. S3. Preparation and characterization of the engineering scaffold.

Fig. S4. Toxicity and degradation evaluation of the engineering scaffold.

Fig. S5. R848@Gel drives the chronic inflammation balance towards the immune activation arm.

Fig. S6. Body weight curves of the 4T1 tumor model were presented.

Fig. S7. The effect of R848&aOX-40@Gel on DCs in the local tumor was shown.

Fig. S8. IFN-γ secretion (A) and TNF-α secretion (B) from re-stimulated splenocytes cultured with X-ray-treated cells were analyzed by ELISA kits.

Fig. S9. R848&aOX40@Gel elicited an antigen-specific immune response.

Fig. S10. Representative scatter plots and gating information derived from analysis of $CD3^{+}$ $CD4^{+}$ or $CD3^{+}$ $CD8^{+}$ T cells in tumors.

Fig. S11. Representative scatter plots and gating information derived from analysis of Treg ($CD4^{+}$ $CD25^{+}$and $Foxp3^{+}$) cells in tumors.

Fig. S12. Representative scatter plots and gating information derived from analysis of effector memory T cells (T_{EM}) and central memory T cells (T_{CM}) in the spleen.

Fig. S13. Representative scatter plots and gating information derived from analysis of immune memory T cells ($CD4^{+}CD44^{+}$ and $CD8^{+}CD44^{+}$) in the spleen.

Table S1. Oxidation degree of A-HA.

Samples ID	Theoretical oxidation degree of A-HA	Actual oxidation degree of A-HA	Average
1	60%	46.16%	
2	60%	48.29%	45.83% ± 2.79%
3	60%	43.04%	

Table S2. List of antibodies used in flow cytometric analysis.

Antibody	Clone	Supplier
CD3- PE/Cyanine7	17A2	BioLegend
CD4-FITC	GK1.5	BioLegend
CD8a-FITC	53-6.7	BioLegend
CD8a-PE	53-6.7	BioLegend
CD11b-PE	M1/70	BioLegend
Gr-1-APC	RB6-8C5	BioLegend
CD25-APC	PC61.5	BioLegend
Foxp3-PE	FJK-16s	BioLegend
Foxp3- PE/Cyanine5	FJK-16s	eBioscience
CD11b-FITC	M1/70	BioLegend
CD11c-FITC	N418	BioLegend
CD40--PE	3/23	BioLegend
CD80-FITC	16-10A1	BioLegend
CD83- PE/Cyanine7	Michel-19	BioLegend
CD86-APC	GL-1	BioLegend
CD44-APC	IM7	BioLegend
CD80-PE	16-10A1	BioLegend
NK1.1- PE/Cyanine5	PK136	BioLegend
CD206-APC	C068C2	BioLegend
F4/80-PE	BM8	BioLegend
CD69-PE	H1.2F3	BioLegend
CD44-FITC	NIM-R8	BioLegend
CD62L-APC	MEL-14	BioLegend

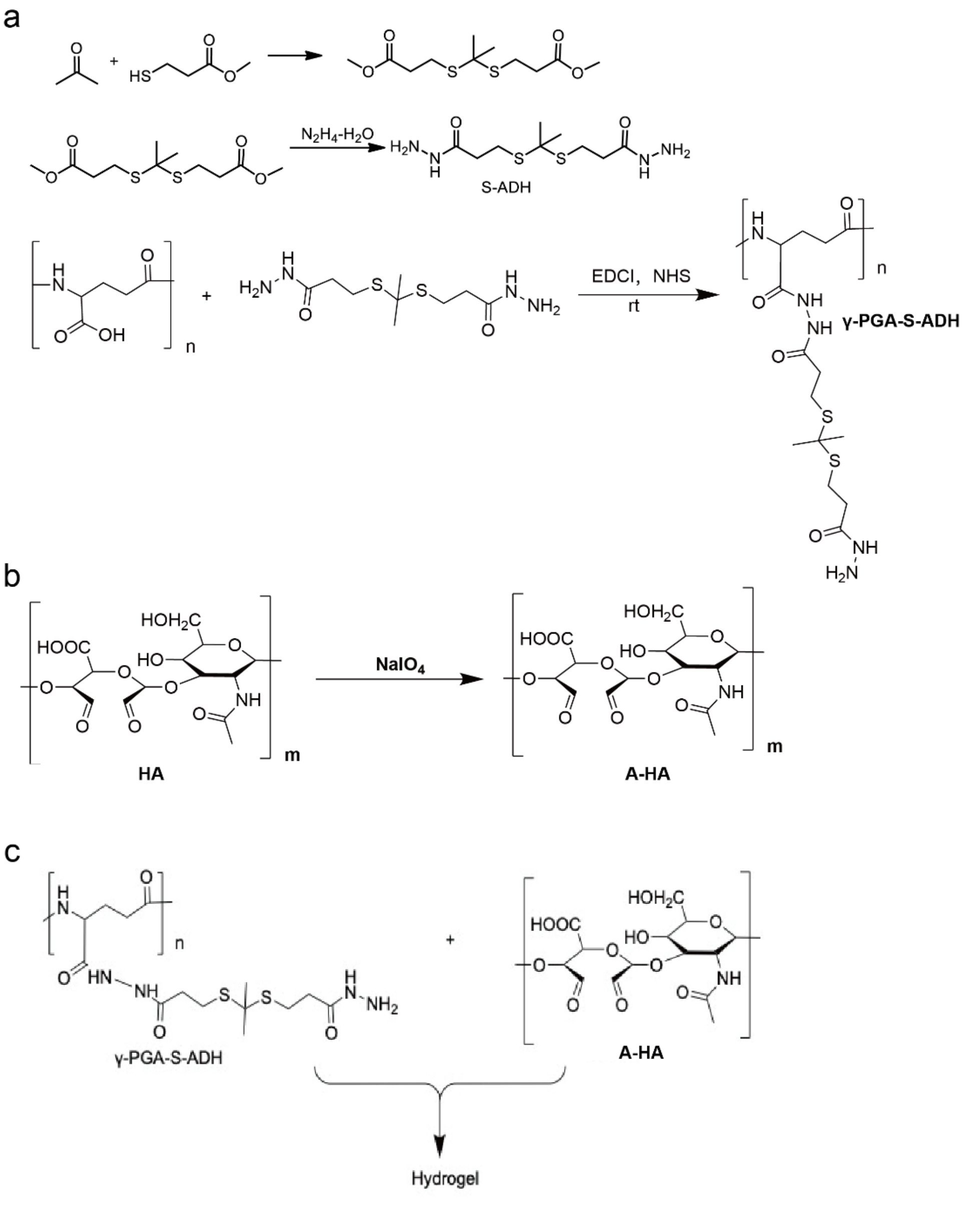

Figure S1. Schematic of the engineering scaffold synthesis. (A) γ-PGA-S-ADH was synthesized via thioketal diol reaction and amidation reaction. (B) A-HA was synthesized by the vicinal diol oxidation. (C) The engineering scaffold was prepared through a chemical crosslinking.

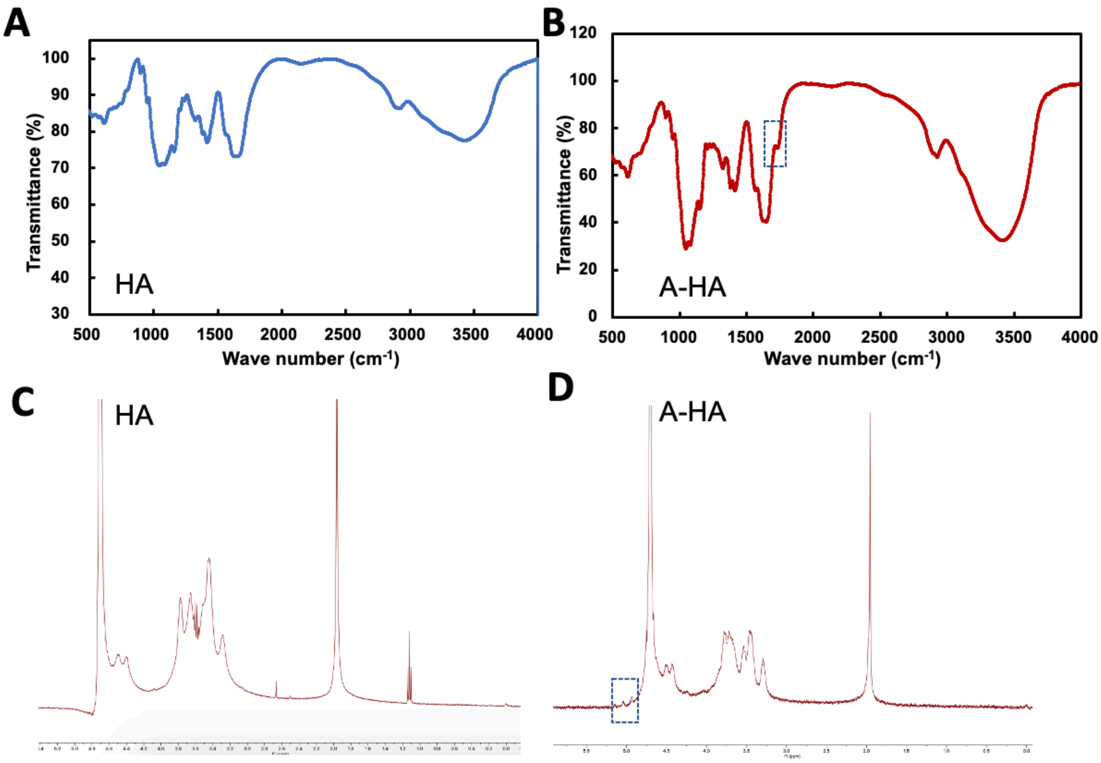

Figure S2. FTIR spectra and ^{1}H-NMR spectra of HA and A-HA. (A) FTIR spectrum of HA, (B) FTIR spectrum of A-HA, (C) ^{1}H-NMR spectrum of HA, (D) ^{1}H-NMR spectrum of A-HA.

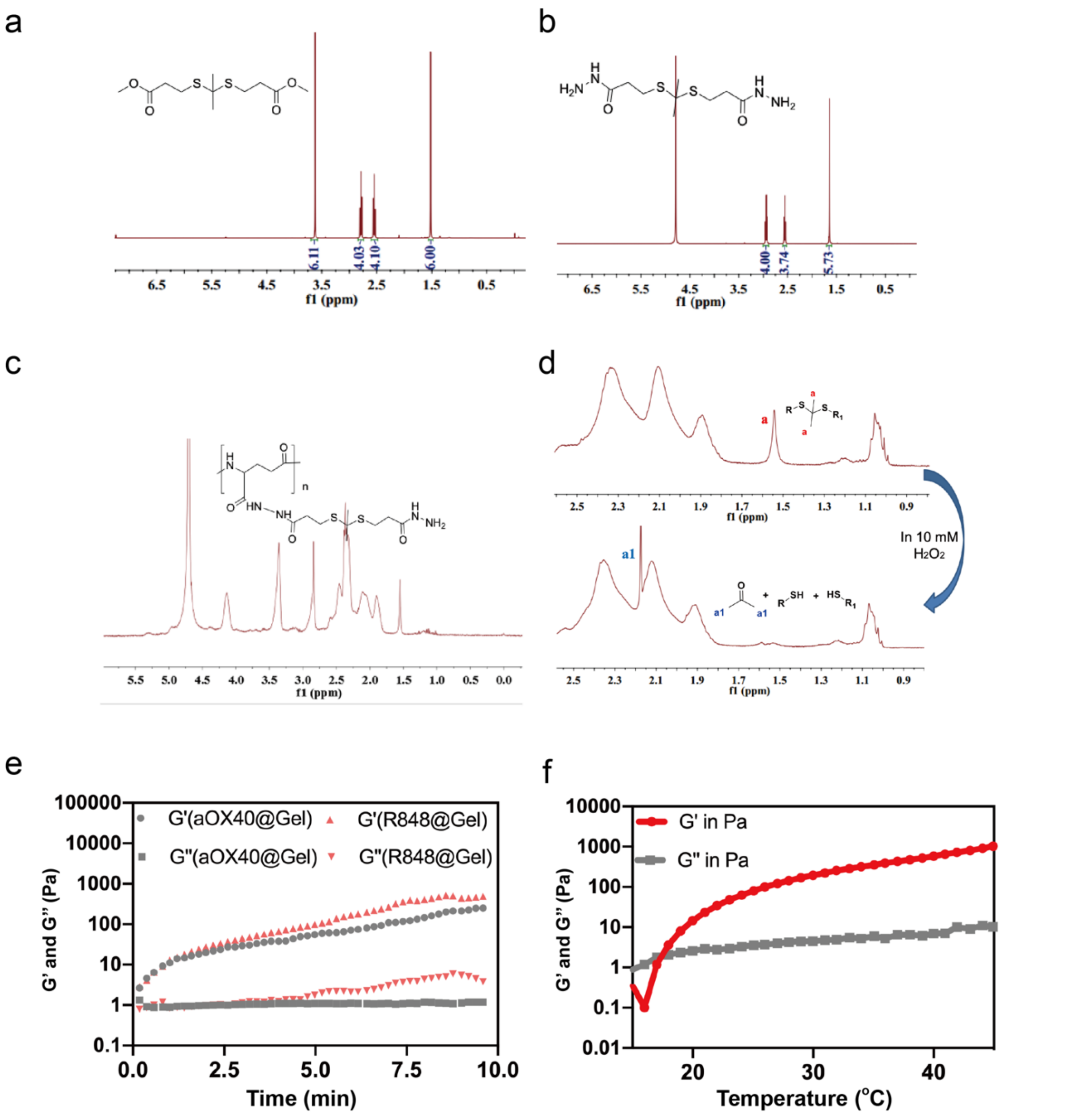

Figure S3. Preparation and characterization of the engineering scaffold. (A-C) *^{1}H*-NMR spectra of ADH (A), S-ADH (B), and γ-PGA-S-ADH (C). (D) ROS response mechanism of the engineering scaffold. γ-PGA-S-ADH/ A-HA scaffold was dissolved in D_2O and D_2O containing 10 mM H_2O_2, then placed at 37 °C for 24 h. Finally, the *^{1}H*-NMR spectrum was shown. (E) Rheological behaviors of the R848@Gel and aOX40@Gel. (F) Rheological behavior of the engineering scaffold with increasing temperature.

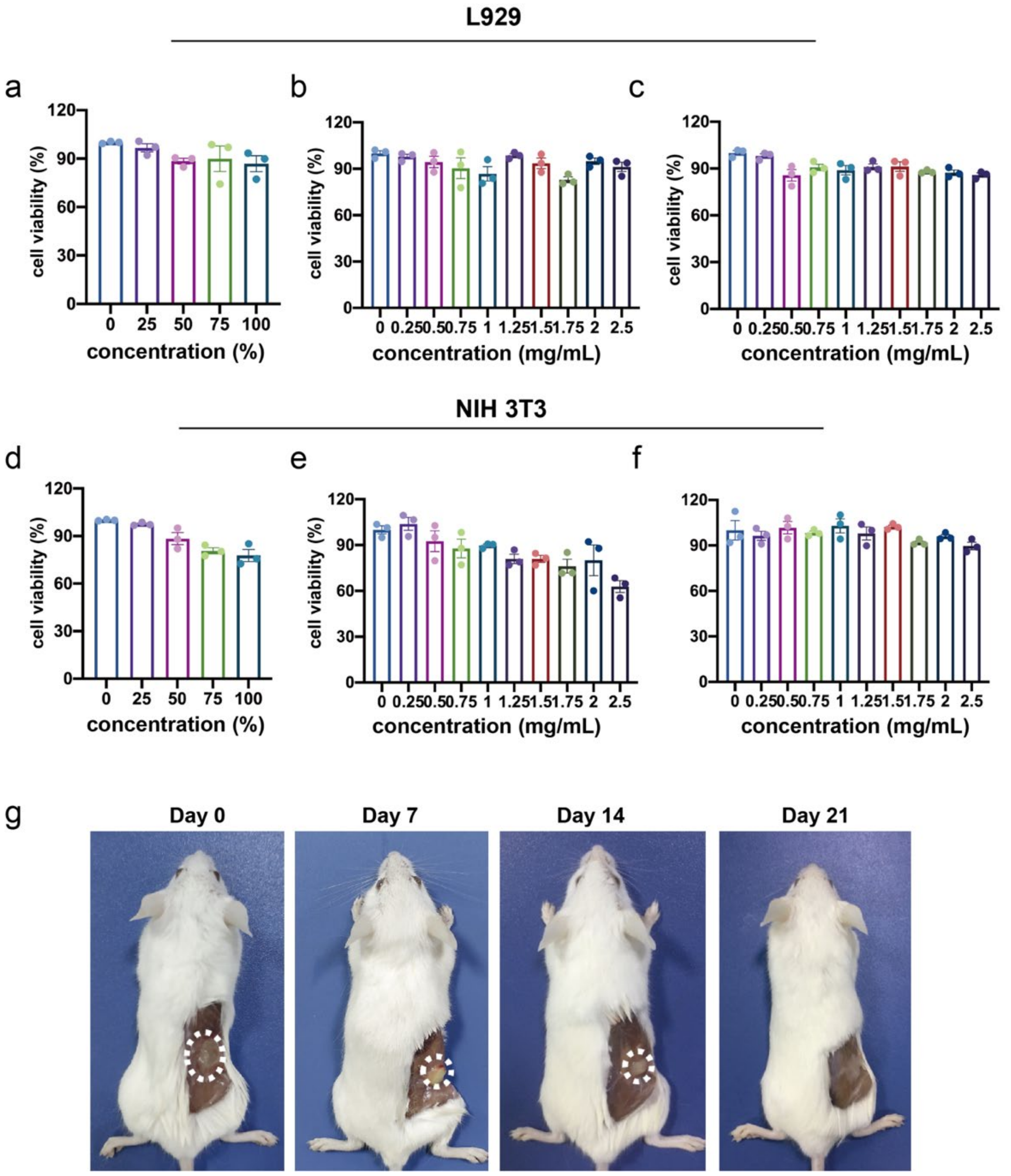

Figure S4. Toxicity and degradation evaluation of the engineering scaffold. (A-C) L929 were incubated with various concentrations of γ-PGA-S-ADH, A-HA, and hydrogel extract for 24 h, respectively, and then detected by MTT. (D-F) L929 were incubated with various concentrations γ-PGA-S-ADH, A-HA, and hydrogel extract for 24 h, respectively, then detected by MTT (n = 3 per group, Data are shown as means ± SEM). (G) 200 uL gel was injected subcutaneously into the right back of the mice. Then the degradation behaviors were observed at particular time points (0, 7, 14, and 21 days).

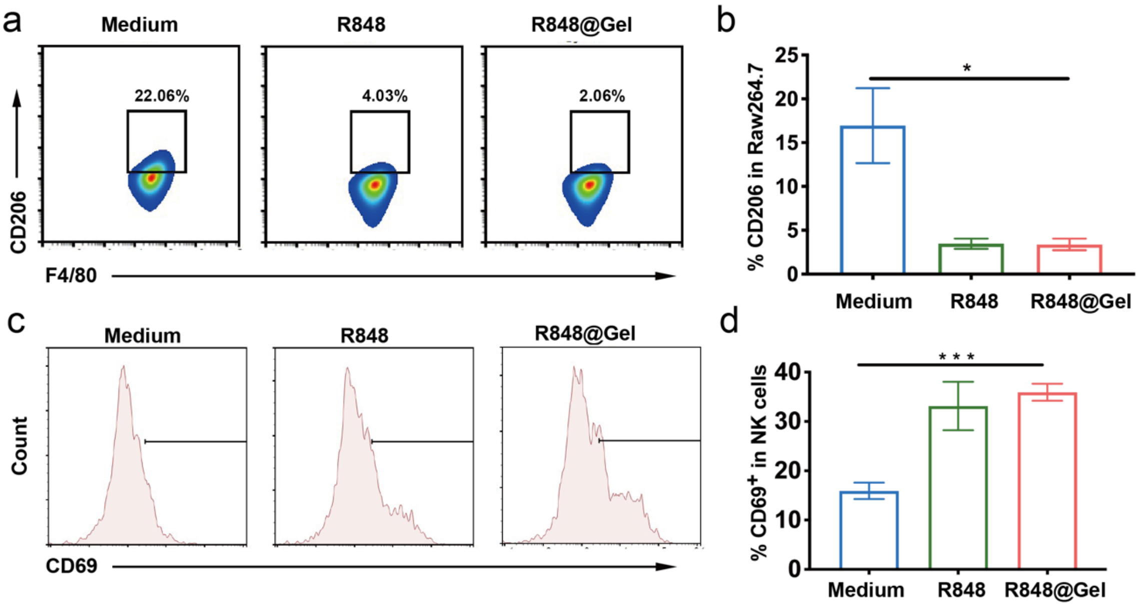

Figure S5. R848@Gel drives the chronic inflammation balance towards the immune activation arm. (A-B) Raw 264.7 cells were cultured in IL-4 medium for 24 h and incubated with free R848 and R848@Gel, and then analyzed by flow cytometry (n = 3 per group). (C-D) R848@Gel induces NK cell activation *in vitro* (n = 3 per group). The data are shown as mean ± SD. Statistical significance was calculated by one-way analysis of variance (ANOVA) with Tukey's post-test. *$P < 0.05$; **$P < 0.01$; ***$P < 0.001$.

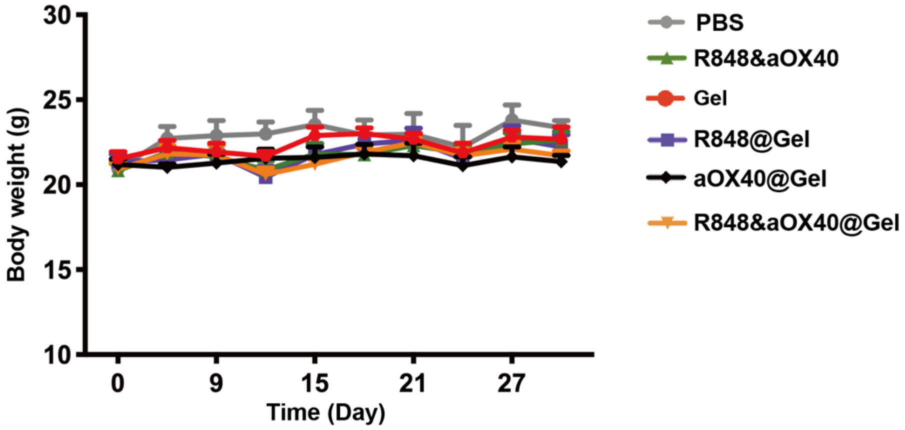

Figure S6. Body weight curves of the 4T1 tumor model were presented.

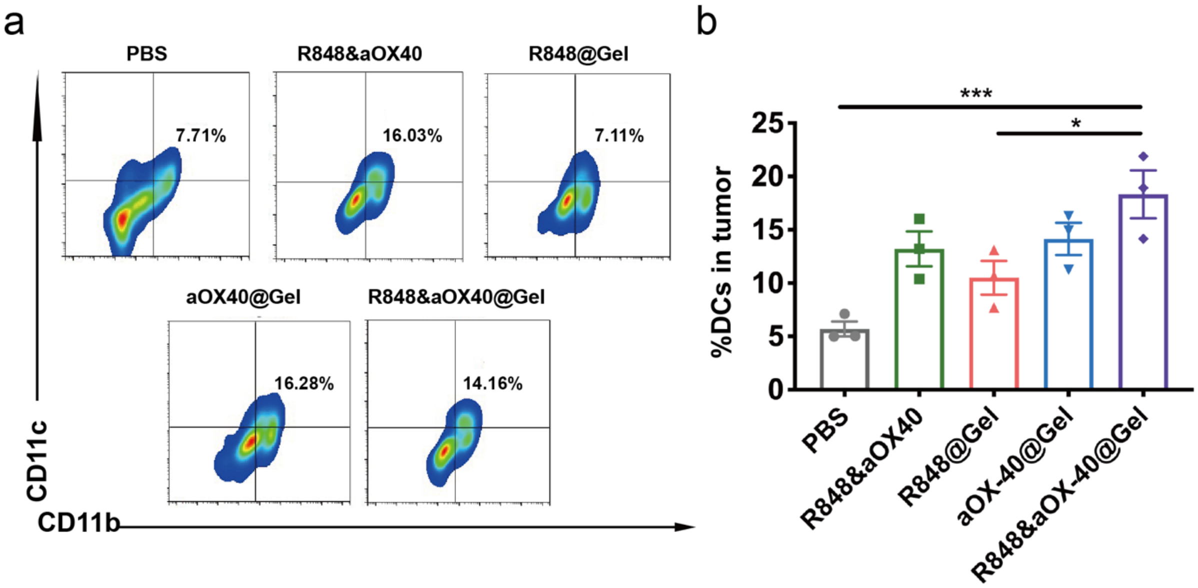

Figure S7. The effect of R848&aOX-40@Gel on DCs in the local tumor was shown (n=3, biologically independent sample). The data are shown as mean ± SEM. Statistical significance was calculated by one-way analysis of variance (ANOVA) with Tukey's post-test. *$P < 0.05$; **$P < 0.01$; ***$P < 0.001$.).

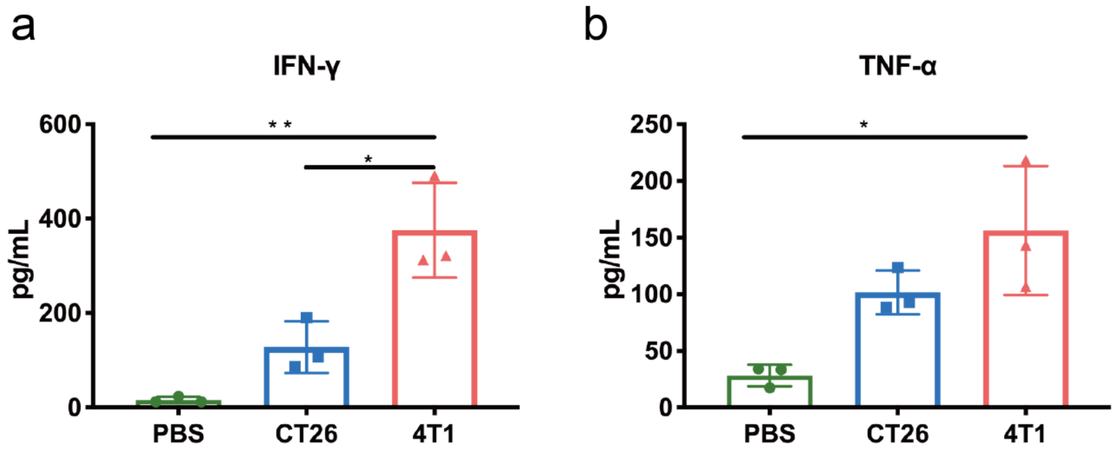

Figure S8. IFN-γ secretion (A) and TNF-α secretion (B) from re-stimulated splenocytes cultured with X-ray-treated cells were analyzed by ELISA kits (n = 3 per group). The data are shown as mean ± SD. Statistical significance was calculated by one-way ANOVA using the Tukey post-test. *P*-value: *, $P < 0.05$; **, $P < 0.01$; ***, $P < 0.001$ and ****, $P < 0.0001$.

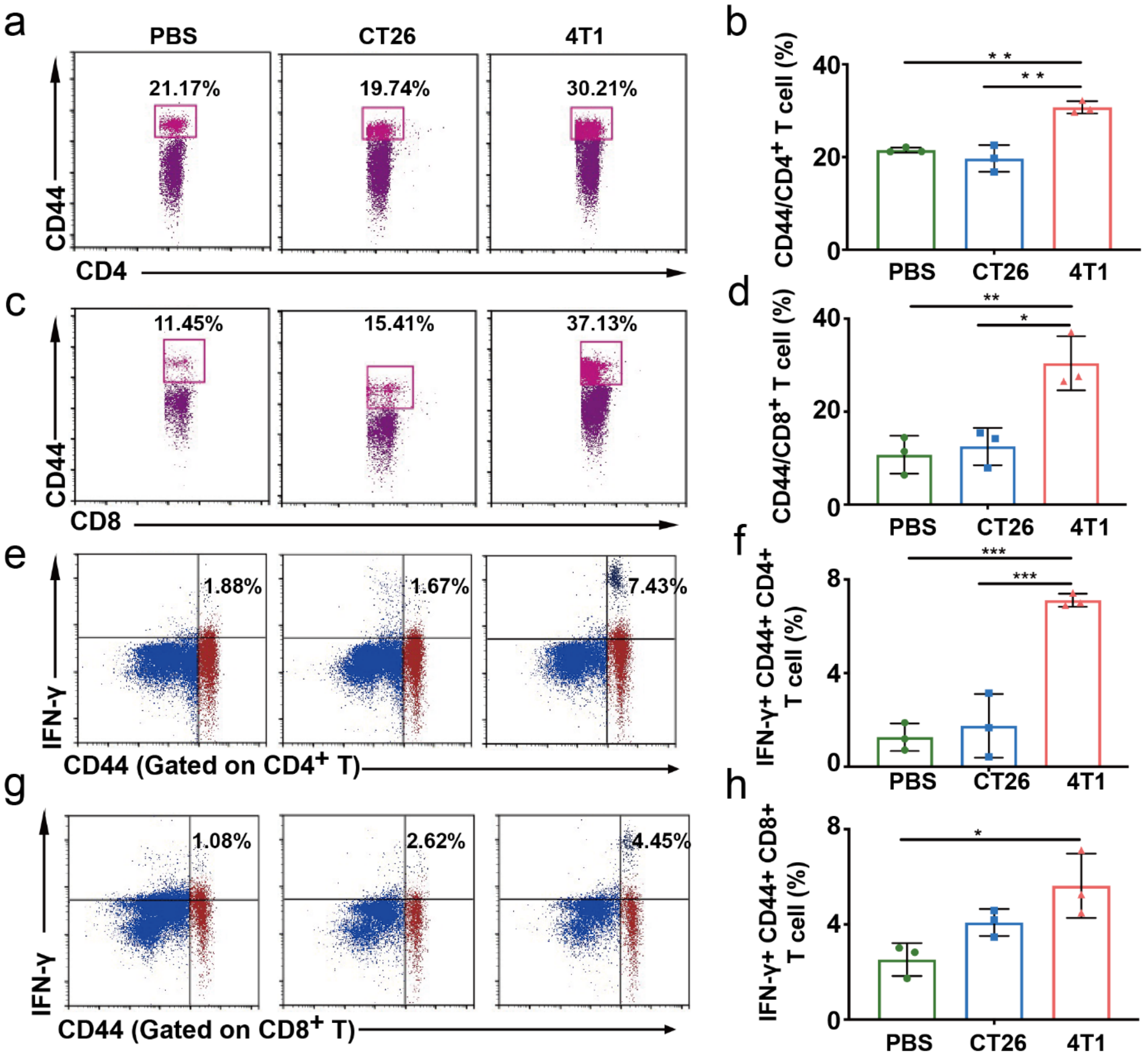

Figure S9. R848&aOX40@Gel elicited an antigen-specific immune response. 4T1-bearing mice were treated with R848&aOX-40@Gel in the local tumor. After 7 days, the single-cell suspensions from the spleen were obtained and co-cultured with X-ray-treated 4T1 and X-ray-treated CT26 tumor cells, respectively. Representative flow cytometric images and percentage of $CD4^+$ $CD44^+$ T cells (A, B), $CD8^+$ $CD44^+$ T cells (C, D), $CD44^+$ IFN-γ^+ T cells gated in CD 4^+ T cells (E, F), and $CD44^+$ IFN-γ^+ T cells gated in CD 8^+ T cells (G, H) were shown (n = 3 per group). The data are shown as mean ± SD. Statistical significance was calculated by one-way ANOVA using the Tukey post-test. *P*-value: *, $P < 0.05$; **, $P < 0.01$; ***, $P < 0.001$ and ****, $P < 0.0001$.

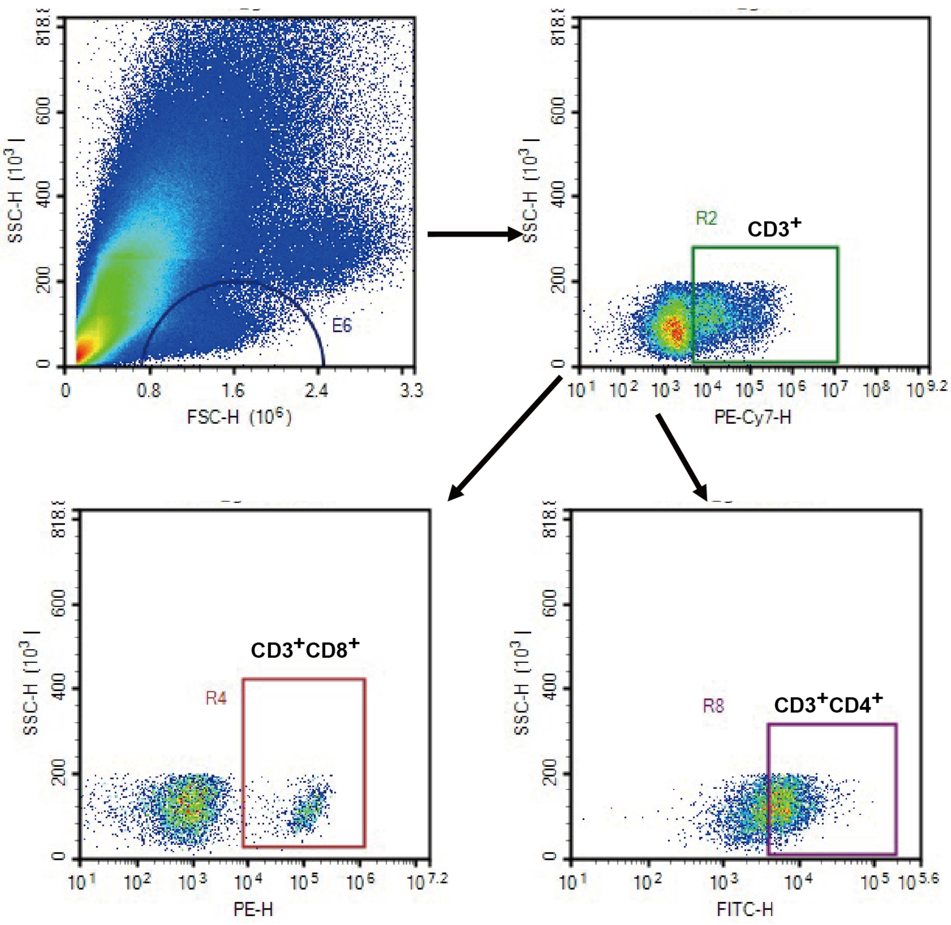

Figure S10. Representative scatter plots and gating information derived from analysis of CD 3^{+} CD 4^{+} and CD3^{+} CD8^{+} T cells in tumors.

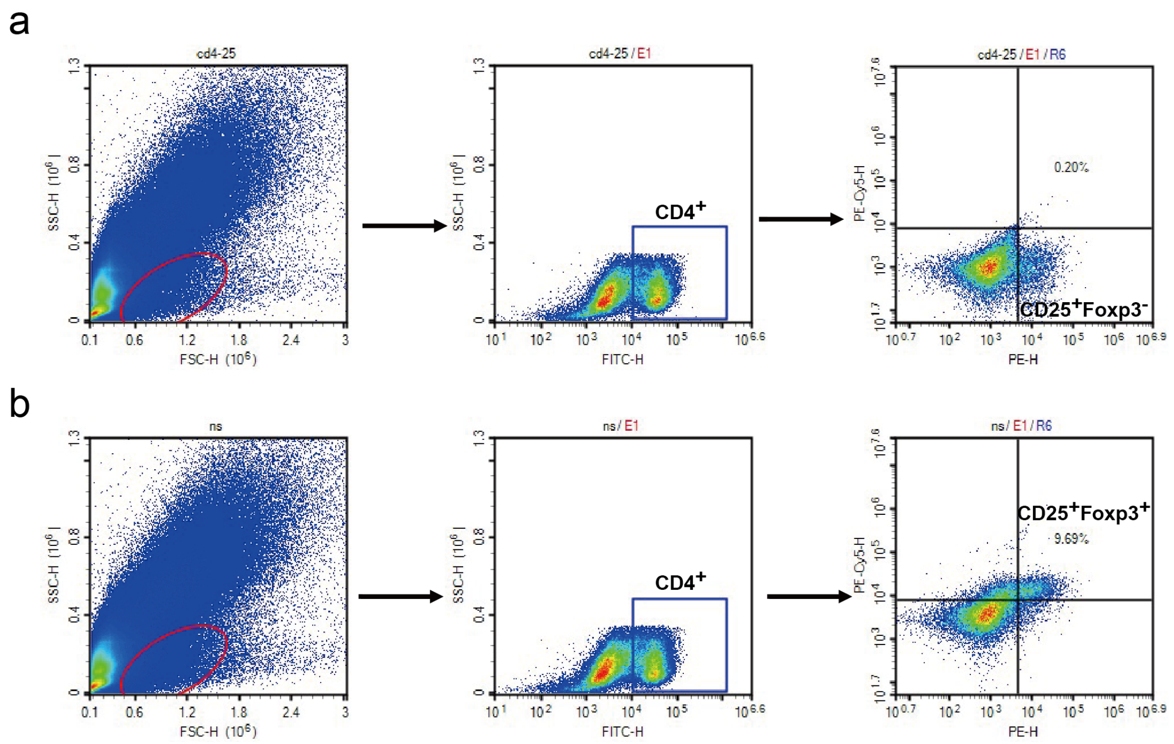

Figure S11. Representative scatter plots and gating information derived from analysis of Treg ($CD4^+$ $CD25^+$ and $Foxp3^+$) cells.

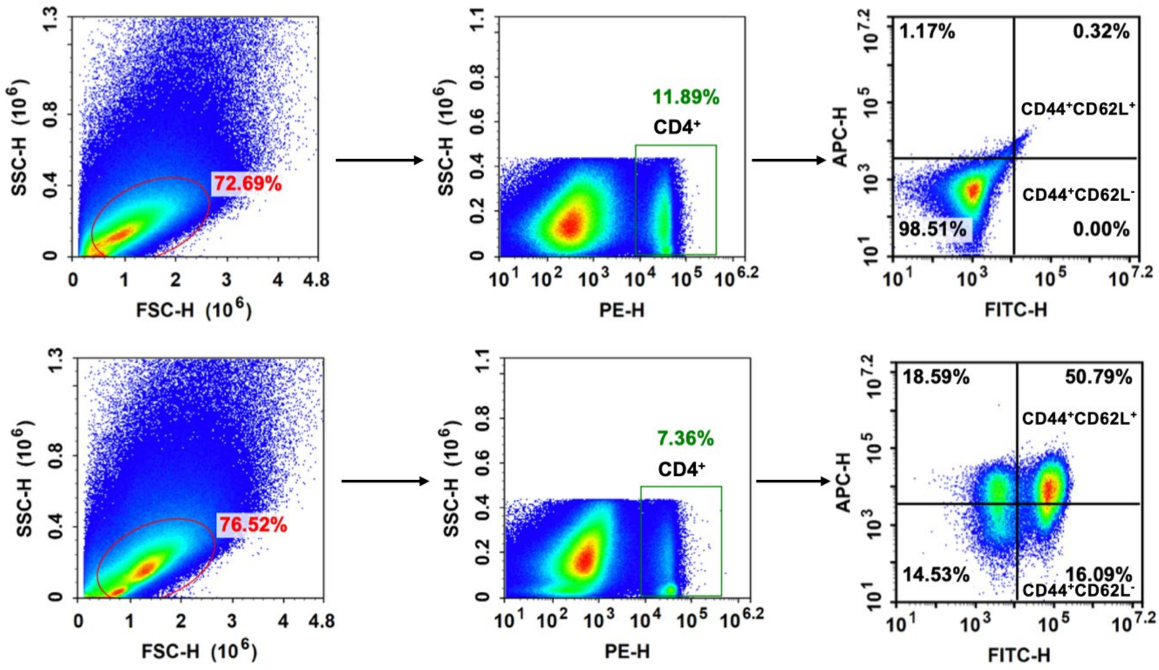

Figure S12. Representative scatter plots and gating information derived from analysis of effector memory T cells (T_{EM}) and central memory T cells (T_{CM}) in the spleen.

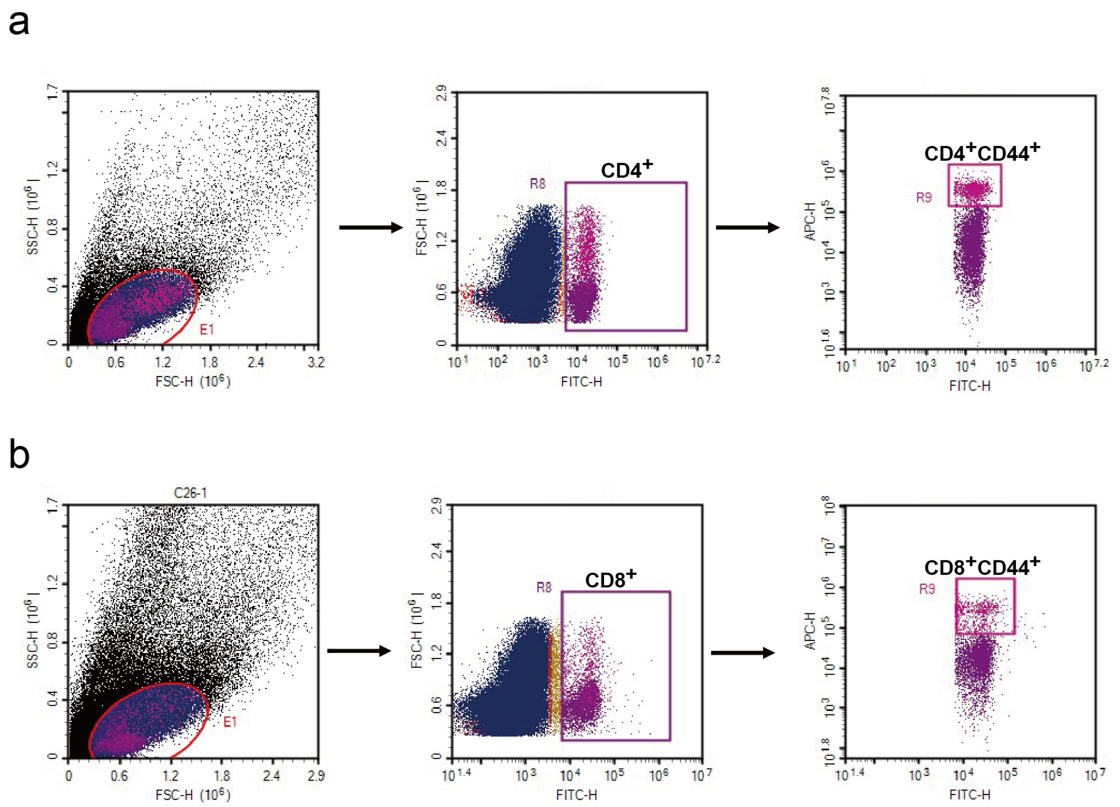

Figure S13. Representative scatter plots and gating information derived from analysis of immune memory T cells ($CD4^+CD44^+$ and $CD8^+CD44^+$) in the spleen.
